# In Silico Design of Antimicrobial Dental Resins Targeting *Streptococcus mutans* Adhesin P1

**DOI:** 10.1002/mbo3.70116

**Published:** 2025-11-17

**Authors:** Ravinder S. Saini, Abdulkhaliq Ali F. Alshadidi, Doni Dermawan, Lujain Ibrahim N. Aldosari, Rayan Ibrahim H. Binduhayyim, Rajesh Vyas, Sunil Kumar Vaddamanu, Mohamed Saheer Kuruniyan, Artak Heboyan

**Affiliations:** ^1^ Department of Allied Dental Health Sciences COAMS King Khalid University Abha Saudi Arabia; ^2^ Applied Biotechnology, Faculty of Chemistry Warsaw University of Technology Warsaw Poland; ^3^ Department of Prosthodontics, College of Dentistry King Khalid University Abha Saudi Arabia; ^4^ Department of Prosthodontics, Faculty of Stomatology Yerevan State Medical University after Mkhitar Heratsi Yerevan Armenia; ^5^ Department of Research Analytics, Saveetha Dental College and Hospitals, Saveetha Institute of Medical and Technical Sciences Saveetha University Chennai India; ^6^ Department of Prosthodontics, School of Dentistry Tehran University of Medical Sciences Tehran Iran

**Keywords:** antimicrobial peptides, dental resin composites, molecular docking, molecular dynamcis, *Streptococcus mutans*, surface protein adhesins

## Abstract

This study explores the potential of incorporating antimicrobial peptides (AMPs) into dental resin composites to enhance resistance against *Streptococcus mutans*, a key contributor to biofilm‐related dental infections through its surface protein adhesins. A comprehensive computational approach was applied to evaluate AMP interactions. Molecular docking was used to assess AMP binding to dental resins, followed by docking the top AMP candidates to *S. mutans* adhesins. The resulting complexes underwent 100 ns molecular dynamics simulations, and binding affinities were refined using MM/PBSA free energy calculations. Several AMPs showed strong binding to dental resins and *S. mutans* adhesins. Pardaxin and tachystatin displayed high affinities for critical adhesion sites. MM/PBSA analysis confirmed strong binding, with tachystatin showing a Δ*G* of –62.03 kcal/mol, significantly better than the standard inhibitor C16G2 (Δ*G* = −33.34 kcal/mol), suggesting enhanced inhibitory potential. Dental composites incorporating specific AMPs show promise in targeting *S. mutans* adhesins and preventing biofilm formation. However, these results are based solely on computational modeling. Experimental validation is essential to confirm biological efficacy, optimize AMP integration into resin formulations, and evaluate safety for potential clinical applications.

## Graphical abstract

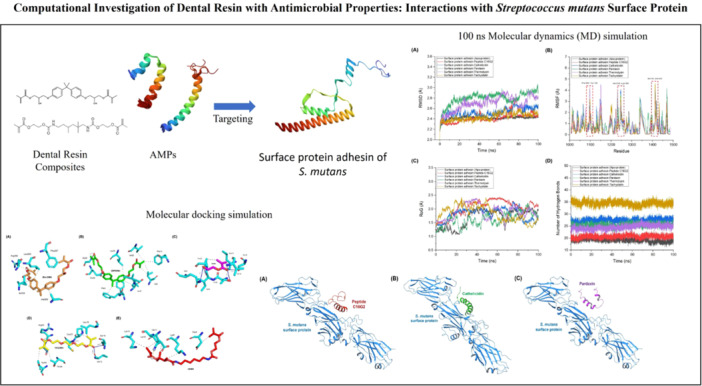

**Abbreviations**
Bis‐GMA: bisphenol A glycidyl methacrylate
EBPADMA: ethoxylated bisphenol A dimethacrylate
HADDOCK: high ambiguity‐driven protein–protein docking
HEMA: 2‐hydroxyethyl methacrylate
ITC: isothermal titration calorimetry
MD: molecular dynamics
MM/PBSA: molecular mechanics/Poisson–Boltzmann surface area
NPT: number of particles, pressure, and temperature
NVT: number of particles, volume, and temperature
OPLS‐AA/L: optimized potentials for liquid simulations
PDB: protein data bank
PRODIGY: PROtein binDIng enerGY prediction
RMSD: root mean square deviations
RMSF: root mean square fluctuation
RoG: radius of gyration
SPCE: single point charge extended
SPR: surface plasmon resonance
TEGDMA: triethylene glycol dimethacrylate
UDMA: urethane dimethacrylate

## 1 Introduction

*Streptococcus mutans* plays a central role in dental caries development due to its ability to form resilient biofilms and adhere to tooth surfaces via surface adhesins (Matsumoto‐Nakano 2018; Lemos et al. 2019). While dental resin composites are widely used in restorative dentistry for their esthetic and mechanical advantages, they are particularly vulnerable to colonization by *S. mutans*, which compromises the longevity of restorations (Lassila et al. 2009; J. Zhang et al. 2024). This study focuses on enhancing the antimicrobial properties of these materials by incorporating antimicrobial peptides (AMPs), which can inhibit *S. mutans* colonization and biofilm formation.

Unlike conventional antimicrobials such as chlorhexidine or silver nanoparticles, which often suffer from cytotoxicity and reduced efficacy over time, AMPs are short peptides with broad‐spectrum antimicrobial activity and relatively low toxicity (Q. Y. Zhang et al. 2021; Makowski et al. 2019). They disrupt bacterial membranes and interfere with biofilm formation, making them attractive for biomedical applications. Prior studies have shown the feasibility of embedding AMPs in dental materials (Montoya et al. 2023; Xie et al. 2020), but their interactions at the molecular level, particularly with key virulence proteins of *S. mutans*, remain underexplored.

Among the virulence factors of *S. mutans*, adhesin P1 (also known as antigen I/II or SpaP) is a major contributor to its pathogenicity (Crowley et al. 1999). This surface protein mediates initial adhesion to tooth surfaces and facilitates biofilm establishment, making it a strategic therapeutic target. Compared with other virulence proteins, P1 plays a more direct role in the early colonization process, which justifies its selection for this study (Manzer et al. 2020; Hu et al. 2022).

This study used computational techniques, including molecular docking and molecular dynamics (MD) simulations, to explore how selected AMPs interact with dental resin composites and the adhesin P1 protein. The goal was to identify promising AMP candidates that could be incorporated into dental materials for improved antimicrobial action against *S. mutans*.

## 2 Methodology

### 2.1 Selection of Dental Resin Composites for Investigation

This stage involved selecting dental resin composites for a detailed computational analysis because of their common use and specific chemical properties relevant to the study goals. These composites were chosen to study their interactions with AMPs and their ability to block surface proteins from *S. mutans*, including adhesin P1, as shown in Table 1. The selection process favored using dental materials in commercial products because of their unique chemical and mechanical features, which affect their interaction with AMPs.

**Table 1:** Chemical structure and density of selected dental resin composites.

Dental resin composite	Chemical structure	Density (g/cm³)
Bisphenol A glycidyl methacrylate (Bis‐GMA)	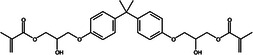	1.16
Ethoxylated bisphenol A dimethacrylate (EBPADMA)	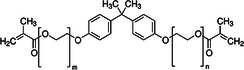	1.12
2‐Hydroxyethyl methacrylate (HEMA)	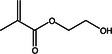	1.03
Triethylene glycol dimethacrylate (TEGDMA)	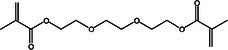	1.07
Urethane dimethacrylate (UDMA)	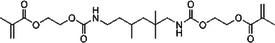	1.11

The dental resin composites used in this study were selected for their widespread application in restorative dentistry and their chemical makeup, which determines their bonding strength, durability, and antimicrobial potential when combined with AMPs. Dental restorations rely on bisphenol A glycidyl methacrylate (Bis‐GMA) because of its high viscosity and strong bonding properties, which provide excellent durability and mechanical performance (Pratap et al. 2019). The inclusion of ethoxylated bisphenol A dimethacrylate (EBPADMA) stems from its lower viscosity compared with Bis‐GMA, which provides better flexibility and application convenience in dental clinics (Skrtic et al. 2006). Dental composite stability in moist oral environments relies on improved tooth structure adhesion enabled by the hydrophilic nature of 2‐hydroxyethyl methacrylate (HEMA) (Van Landuyt et al. 2008). The composite materials received improved mechanical strength by incorporating triethylene glycol dimethacrylate (TEGDMA), a diluent monomer that enhances cross‐linking (Comeau and Willett 2020). Urethane dimethacrylate (UDMA) was selected because its optimal mechanical attributes and flexibility make it suitable for multiple restorative uses (Yang et al. 2024). The selected dental composites provided a foundational basis to study their chemical composition interactions with AMPs and their capability to boost the antimicrobial properties of dental materials. This study investigated the relationship between dental resin and the surface proteins of *S. mutans* to develop antimicrobial dental composites that stop biofilm formation and reduce the occurrence of dental caries.

### 2.2 Selection of AMPs

To ensure reliable computational results, this study selected 30 AMPs based solely on the availability of experimentally resolved three‐dimensional (3D) structures from the Protein Data Bank (PDB), as accurate atomic‐level data are crucial for molecular docking and dynamics. While this criterion limited the peptide pool, it allowed consistent structural input without relying on potentially inaccurate predicted models. All 30 PDB‐derived AMPs were docked against each of the five resin monomers to provide a comprehensive interaction assessment. Peptides were further confirmed as “peptides” based on the Structural Classification of Proteins to maintain relevance to antimicrobial functions. Structures were limited to high‐resolution models (0.5–2.5 Å) to improve simulation reliability. Active sites on each peptide were identified using CASTp 3.0 (W. Tian et al. 2018), aiding in prioritizing key residues during interaction analysis. Supporting Information Data 1 contains the complete list of AMPs, including PDB IDs and active residues. Peptide selections were based on both antimicrobial relevance and structural availability. For instance, aurein was selected for its broad‐spectrum membrane‐disrupting ability, cathelicidin for its dual antimicrobial and immunomodulatory roles, and hepcidin for its effectiveness across bacterial types via iron‐regulating activity. Pardaxin and tachystatin were included due to their membrane‐targeting and cell wall‐specific mechanisms. These peptides were evaluated for interaction with dental resin composites and their potential to inhibit *S. mutans* adhesin P1, a critical factor in dental biofilm initiation (Table 2).

**Table 2:** Selected antimicrobial peptides (AMPs) based on the employed criterion.

AMP	PDB ID	Active site (number of residues)	Reference
Aurein	1VM5	1, 2, 5	Wang et al. (2005)
Beta‐defensin 2	1FD4	6, 7, 9, 10, 11, 12	Hoover et al. (2000)
Bombinin	2AP7	1, 3, 4, 6, 7	Zangger et al. (2008)
Cathelicidin	2K6O	1, 4, 5, 8	Wang (2008)
Cecropin	1D9J	7, 9, 10, 11, 12	Oh et al. (1999)
Chim2	8EB1	10, 11, 14, 15	Viana de Freitas et al. (2023)
Dermcidin	2NDK	18, 19, 22, 25, 26, 29	Nguyen et al. (2017)
Esculentin	5XDJ	2, 3, 6	Loffredo et al. (2017)
Exendin‐4	3C59	26, 27, 28, 29, 32, 33	Runge et al. (2008)
Hepcidin	3H0T	13, 14, 16, 18, 19, 20, 21, 22	Jordan et al. (2009)
Hs05	6VLA	5, 8, 9	Mariano et al. (2021)
Indolicidin	1HR1	9, 10, 11, 12, 13	Friedrich et al. (2001)
Lactoferrin	1LFC	1, 23, 25	Hwang et al. (1998)
Lavracin	2N8D	1, 3, 4, 6	Pillong et al. (2017)
Magainin	2MAG	1, 2, 6, 9, 17, 21	Gesell et al. (1997)
Melittin	2MLT	13, 16, 17, 20	Terwilliger et al. (1982)
Microcin J25	4CU4	9, 10, 19, 20, 21	Mathavan et al. (2014)
Nisin	1WCO	9, 12, 17, 19, 20, 21	Hsu et al. (2004)
Pardaxin	2KNS	2, 3, 5, 6, 9, 15, 22, 23, 26, 27, 29, 30, 33	Bhunia et al. (2010)
Piscidin	6PEZ	3, 4, 7	Comert et al. (2019)
Pleurocidin	2LS9	10, 13, 14, 17, 20, 23, 24	Amos et al. (2016)
Polyphemusin I	1RKK	7, 9, 12, 14	Powers et al. (2004)
Protegrin‐1	1PG1	5, 6, 7, 14, 15, 16	Fahrner et al. (1996)
PvHCt	2N1C	14, 15, 16, 17, 18, 19, 20, 22, 23	Petit et al. (2016)
Subtilisin A	1PXQ	1, 4, 5, 7, 9, 10, 24, 25, 29, 30, 33	Kawulka et al. (2004)
Tachyplesin‐1	2RTV	1, 2, 3, 16, 17	Kushibiki et al. (2014)
Tachystatin	1CIX	5, 11, 12, 15, 18, 22, 23, 29	Fujitani et al. (2002)
Temporin‐L	6GS5	4, 7	Manzo et al. (2019)
Thanatin	8TFV	11, 13, 16, 17, 18	Mandard et al. (1998)
Thermolysin	6FHP	258, 263, 267, 305, 306, 309, 310	Fiebig et al. (2018)

### 2.3 Molecular Docking Simulations

Molecular docking simulations examined how AMPs interact with dental resin composites and their potential binding to the *S. mutans* surface protein adhesin. This study aimed to determine AMPs' binding mechanisms to dental resin materials and the bacterial protein adhesin P1, which facilitates biofilm formation and bacterial adhesion on dental surfaces. High Ambiguity‐Driven protein–protein DOCKing (HADDOCK) version 2.4 (Dominguez et al. 2003) was employed due to its reliability in modeling protein and peptide interactions with defined active sites. The process is divided into two stages. The initial phase of the docking simulations involved inserting AMPs into dental resin composites to identify the peptides that demonstrated optimal binding interactions with the resin matrix. This analysis aimed to determine if the AMPs would blend properly into the resin while maintaining their mechanical strength and if they would preserve their antimicrobial effectiveness when embedded in the dental substance.

AMPs that showed superior results in resin‐composite docking simulations underwent additional docking tests against the surface protein adhesin of *S. mutans* named adhesin P1. The X‐ray crystal structure for adhesin P1 (PDB ID: 4TSH, Heim et al. 2014), with a resolution of 2.00 Å, was downloaded from the PDB. The CASTp 3.0 analysis showed that the active site of the protein consists of essential residues 1023, 1155, 1156, 1184, 1189, 1191, 1192, 1212, 1213, 1215, 1265, 1267, 1309, 1320, 1321, 1322, 1327, and 1329, which are crucial for its adhesive function. This study focused on studying AMP interactions with active site residues and evaluating their effectiveness in blocking adhesin P1 function to prevent bacterial adhesion and biofilm development on dental surfaces. For all docking runs, HADDOCK was configured to apply semi‐flexible docking with flexible residues automatically selected around active and passive interfaces. Default water refinement was applied in the final refinement stage to enhance interaction realism.

To benchmark the efficacy of these AMPs, peptide C16G2 (sequence: TFFRLFNRSFTQALGKGGGKNLRIIRKGIHIIKKY) served as the standard reference AMP in this study because it is a known inhibitor of adhesin P1. This peptide has demonstrated the potential to decrease *S. mutans* adhesion and is an ideal reference for selected AMPs (Kaplan et al. 2011; Namburu et al. 2022). The simulations compared the docking results between C16G2 and AMPs to discover peptides that best disrupt bacterial adhesion. Docking results were ranked based on HADDOCK scores, which reflect a weighted combination of van der Waals, electrostatics, desolvation energy, and restraint violation energies. PROtein binDIng enerGY prediction (PRODIGY) (Grassmann et al. 2024) used a sophisticated methodology to determine the binding affinities of AMP–resin and AMP–protein complexes by analyzing structural interactions. This stage is crucial for implementing a ranking system that determines the best complexes to advance into later experimental stages. In this study, binding strength was evaluated using free binding energy (Δ*G*) values predicted by PRODIGY, where values below −7 kcal/mol were interpreted as indicative of strong binding affinity, consistent with established benchmarks for protein–peptide interactions. The simulation was run on a high‐performance computing workstation with an Intel Core i7‐12650H processor and an NVIDIA RTX 4060 graphics card with 16 GB DDR5 RAM. The system configuration allows for efficient data processing of large data sets and detailed structural models, leading to precise and dependable results.

### 2.4 MD Simulations

Peptide–protein interactions between AMPs and the surface protein adhesin of *S. mutans* were studied using MD simulations to analyze complex stability and dynamic behavior. We conducted the MD simulations with GROMACS 2022.5 (Pronk et al. 2013), a well‐regarded MD software that models biomolecular interactions efficiently and accurately. The Optimized Potentials for Liquid Simulations (OPLS‐AA/L) force fields were selected for accurate modeling of molecular interactions because of their high suitability for peptide–protein complex simulations (Alotaiq et al. 2024; Dermawan and Alotaiq 2025a; Doni Dermawan et al. 2024). The simulation system was built using a cubic box with a 1.0 nm buffer distance and solvated using the single point charge extended water model to simulate realistic solvation effects. Counterions (Na^+^ or Cl^−^) were added to neutralize the total charge of the system, mimicking physiological conditions (Yuet and Blankschtein 2010). The steepest descent energy minimization was performed until the system reached a maximum force below 1000 kJ/mol/nm, eliminating steric clashes.

The system then underwent a two‐step equilibration process. First, a 100‐ps number of particles, volume, and temperature equilibration was performed at 300 K, using the V‐rescale thermostat to stabilize the temperature. Second, a 100‐ps number of particles, pressure, and temperature equilibration was carried out at 1 bar pressure using the Parrinello–Rahman barostat, ensuring pressure stabilization and density convergence. The integration time step was set to 2 fs, and library of integrated network‐based cellular signatures constraints were applied to all bonds involving hydrogen atoms to enable the larger time step. Following equilibration, the production MD simulation was run for 100 ns, which is commonly sufficient to observe convergence and dynamic stabilization for protein–peptide complexes in systems of this scale. This timescale was selected based on prior studies demonstrating equilibrium and structural stability of similar AMP–protein systems within 80–100 ns.

Structural analyses included calculations of Root Mean Square Deviation (RMSD) and Root Mean Square Fluctuation (RMSF) to monitor complex stability and flexibility, while the radius of gyration (RoG) was used to evaluate structural compactness. We also analyzed hydrogen bonding patterns and total potential energy trends to further assess binding stability. For molecular mechanics/Poisson–Boltzmann surface area (MM/PBSA) calculations, snapshots were extracted every 100 ps during the final 20 ns of the production run after verifying that the system had reached equilibrium based on RMSD plateauing. These snapshots were used to estimate the binding free energies of the AMP–adhesin complexes, offering insight into interaction strengths under physiological conditions. Visualization and residue interaction analyses were performed using python molecular (Schrödinger 2020) and UCSF Chimera version 1.18 (Pettersen et al. 2004), allowing us to manually inspect key interfacial residues and validate binding interface observations from MD outputs.

### 2.5 MM/PBSA Calculations

Researchers have used the MM/PBSA method to evaluate how peptides interact with the adhesin protein on the surface of *S. mutans*. MD simulations generated multiple protein conformations from which representative snapshots were selected for further energy analysis (S. Tian et al. 2014). A total of 200 snapshots were extracted at 100 ps intervals from the final 20 ns of the production MD simulation for MM/PBSA calculations. The energy calculations for each snapshot included gas‐phase energy measurements (electrostatic and van der Waals interactions) and solvation energy components (polar and nonpolar contributions) using a continuum solvent model. However, entropy contributions (−*T*Δ*S*) were not explicitly included in this study, as normal mode analysis for entropy estimation is computationally demanding and less reliable for short peptides. While we acknowledge that omitting entropy reduces the thermodynamic completeness of MM/PBSA, the enthalpic component (Δ*H*) remains a useful comparative metric for ranking peptide binding affinities across systems. The energy calculations for each snapshot included gas‐phase energy measurements and solvation energy using a continuum solvent model and entropy estimates. The binding free energy of the peptide–protein complex was determined by a combination of individual energy components (Z. Yuan et al. 2024; Rifai et al. 2020). The gmx_MMPBSA module from the GROMACS simulation suite performed MM/PBSA calculations (Valdés‐Tresanco et al. 2021; Miller et al. 2012). This tool's known precision and efficiency for calculating binding free energies in biomolecular complexes make it well‐suited for analyzing AMP interactions with protein adhesin. Scientists frequently use the MM/PBSA method to predict binding free energies, which helps researchers explore the energy landscapes of peptide–protein interactions (Panday and Alexov 2022; Saini et al. 2024a). The MM/PBSA binding free energy calculation is based on the following equation:

$$\Delta G_{\mathrm{binding}}=\Delta G_{\mathrm{complex}}-\Delta G_{\mathrm{peptide}}-\Delta G_{\mathrm{protein}},$$

where

Δ*G*_binding_: The binding free energy associated with forming the protein–protein complex.

Δ*G*_complex_: The free energy of the fully solvated protein–protein complex.

Δ*G*_peptide_: The free energy of the peptide in its solvated state when unbound.

Δ*G*_protein_: The free energy of protein in its solvated state when unbound.

The binding free energy calculation involved subtracting the total free energy of the individual, unbound components from that of the complex. Although entropy was not included, this computational analysis still revealed energy changes during complex formation, offering valuable insight into the relative strength and stability of AMP–adhesin interactions.

## 3 Results

### 3.1 Molecular Docking Simulations of AMPs and Dental Resin Composites

Molecular docking simulations provided precise data regarding how the AMP–resin composite complexes interacted and their energetic profiles. This analysis identified the best component mixtures that could be used in practical dental material applications. AMPs exhibit diverse interaction mechanisms with resin composites, as demonstrated by their hydrophobic interactions, hydrogen bonds, and salt bridges. These components provide essential stability and binding strength to composite complexes through their interactions.

**Figure 1:**
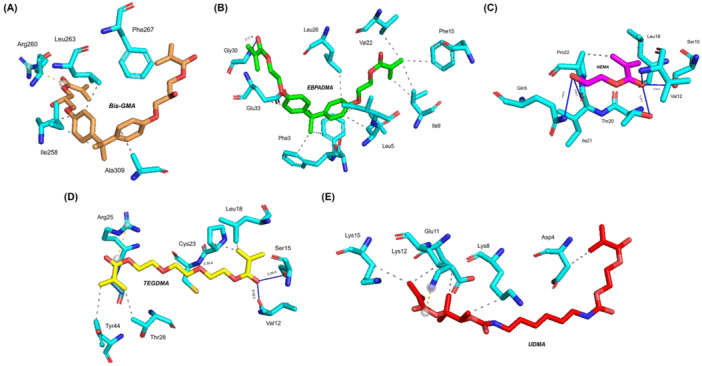
Three‐dimensional perspective of the most favorable complexes for each dental resin composite. (A) Bis‐GMA:thermolysin complex, (B) EBPADMA:pardaxin complex, (C) HEMA:tachystatin complex, (D) TEGDMA:tachystatin complex, and (E) UDMA:cathelicidin complex. Bis‐GMA, bisphenol A glycidyl methacrylate; EBPADMA, ethoxylated bisphenol A dimethacrylate; HEMA, 2‐hydroxyethyl methacrylate; TEGDMA, triethylene glycol dimethacrylate; UDMA, urethane dimethacrylate.

The optimal complexes determined for each dental‐resin composite are represented by the 3D models shown in Figure 1. For instance, the Bis‐GMA:thermolysin complex maintains its stability through five hydrophobic interactions and one salt bridge that creates strong connections between its parts. The EBPADMA:pardaxin complex demonstrated strong specific binding affinity by forming 11 hydrophobic interactions along with a hydrogen bond with Gly30 at a 2.11‐Å distance. Similarly, the HEMA:tachystatin complex established two hydrophobic contacts and five hydrogen bonds with Gln5, Val12, Ser15, Thr20, and Ile21 and showed bond lengths between 1.99 and 3.00 Å, which confirms a stable binding interface. Furthermore, the TEGDMA:tachystatin complex formed four hydrophobic interactions with one salt bridge and four hydrogen bonds with Val12, Ser15, Cys23, and Gly26, which measured bond distances between 2.29 and 2.77 Å. The observed interaction pattern indicates stability and strength, which may improve material performance. The UDMA:cathelicidin complex demonstrated four hydrophobic interactions and one salt bridge, which stated a stable interaction that may enhance the antimicrobial properties of the material. Table 3 summarizes the molecular docking simulation results, which show binding energy values and various energy components measured in kilocalories per mole (kcal/mol). The detailed results offer a quantitative evaluation of the interactions, which enables researchers to compare the stability and affinity across AMP–resin composite complexes. The full molecular docking simulation results are documented in Supporting Information Data 2.

**Table 3:** Molecular docking outcomes for the five best‐performing dental resin composites and antimicrobial peptides (AMPs) complexes.

Resin composite: AMP complex	HADDOCK score (a.u.)	Binding energy (kcal/mol)	van der Waals energy	Electrostatic energy	Desolvation energy	RMSD
*Bis‐GMA complexes*						
Bis‐GMA:thermolysin	−34.1 ± 1.5	−8.08	−21.0 ± 1.2	−31.2 ± 7.8	−10.4 ± 0.4	0.3 ± 0.0
Bis‐GMA:tachystatin	−38.8 ± 1.6	−7.98	−32.7 ± 0.6	−17.0 ± 4.0	−6.1 ± 0.8	0.2 ± 0.1
Bis‐GMA:nisin	−35.6 ± 0.7	−7.60	−25.5 ± 1.2	−78.7 ± 9.4	−2.3 ± 0.5	1.6 ± 0.1
Bis‐GMA:pardaxin	−48.7 ± 0.9	−7.59	−29.0 ± 1.1	−1.9 ± 2.1	−20.0 ± 0.8	0.6 ± 0.1
Bis‐GMA:bombinin	−27.4 ± 1.0	−7.57	−19.2 ± 0.5	−67.5 ± 4.1	−1.5 ± 0.4	0.3 ± 0.2
*EBPADMA complexes*						
EBPADMA:pardaxin	−41.9 ± 1.9	−8.14	−27.0 ± 2.3	−30.1 ± 10.6	−12.8 ± 1.5	0.5 ± 0.3
EBPADMA:tachystatin	−35.8 ± 1.0	−7.93	−27.6 ± 1.2	−15.3 ± 3.6	−7.1 ± 1.0	0.3 ± 0.2
EBPADMA:thermolysin	−33.0 ± 0.6	−7.87	−20.5 ± 0.3	−20.9 ± 6.0	−10.5 ± 0.8	0.4 ± 0.0
EBPADMA:pleurocidin	−45.1 ± 0.9	−7.64	−31.2 ± 0.5	−30.1 ± 11.3	−11.1 ± 0.8	0.8 ± 0.2
EBPADMA:protegrin−1	−28.4 ± 0.7	−7.46	−23.2 ± 0.9	−54.5 ± 7.7	0.2 ± 0.3	0.4 ± 0.1
*HEMA complexes*						
HEMA:tachystatin	−20.7 ± 0.2	−6.24	−17.0 ± 0.2	−27.1 ± 0.8	−1.0 ± 0.1	0.1 ± 0.1
HEMA:Chim2	−19.2 ± 0.4	−6.17	−13.2 ± 0.7	−35.0 ± 3.2	−2.6 ± 0.5	0.3 ± 0.2
HEMA:cecropin	−18.6 ± 1.0	−6.10	−13.8 ± 0.7	−37.3 ± 7.8	−1.0 ± 0.4	0.4 ± 0.2
HEMA:thermolysin	−14.5 ± 0.6	−6.03	−8.2 ± 0.3	−1.5 ± 0.6	−6.3 ± 0.3	0.2 ± 0.1
HEMA:subtilisin A	−15.2 ± 2.4	−5.98	−13.8 ± 0.5	−26.1 ± 4.6	−0.2 ± 0.3	0.8 ± 0.1
*TEGDMA complexes*						
TEGDMA:tachystatin	−31.9 ± 1.3	−6.90	−23.8 ± 1.2	−46.4 ± 7.1	−3.8 ± 0.1	0.3 ± 0.0
TEGDMA:thermolysin	−25.9 ± 1.1	−6.81	−15.9 ± 0.5	−19.5 ± 7.2	−8.2 ± 0.2	0.2 ± 0.1
TEGDMA:beta‐defensin 2	−23.7 ± 0.5	−6.77	−14.5 ± 0.8	−68.0 ± 3.4	−2.6 ± 0.2	0.2 ± 0.0
TEGDMA:pardaxin	−30.4 ± 1.1	−6.59	−17.0 ± 0.9	−4.5 ± 2.1	−13.6 ± 1.1	0.4 ± 0.2
TEGDMA:PvHCt	−25.5 ± 0.8	−6.59	−15.7 ± 0.9	−33.5 ± 4.5	−8.7 ± 0.6	0.7 ± 0.1
*UDMA complexes*						
UDMA:cathelicidin	−30.6 ± 0.8	−7.42	−20.1 ± 0.5	−96.0 ± 8.6	−1.0 ± 0.3	2.0 ± 0.0
UDMA:pardaxin	−38.6 ± 0.6	−7.09	−22.0 ± 1.2	−5.9 ± 5.2	−16.5 ± 1.7	0.5 ± 0.1
UDMA:pleurocidin	−34.4 ± 1.3	−6.99	−21.2 ± 0.6	−31.9 ± 12.2	−10.8 ± 1.1	1.4 ± 0.1
UDMA:Chim2	−31.3 ± 2.7	−6.86	−16.4 ± 1.8	−26.9 ± 4.0	−12.4 ± 0.6	1.7 ± 0.1
UDMA:subtilisin A	−28.0 ± 0.9	−6.85	−22.0 ± 0.6	−47.3 ± 5.2	−1.8 ± 0.3	1.3 ± 0.0

Abbreviations: Bis‐GMA, bisphenol A glycidyl methacrylate; EBPADMA, ethoxylated bisphenol A dimethacrylate; HADDOCK, High Ambiguity‐Driven protein–protein DOCKing; HEMA, 2‐hydroxyethyl methacrylate; RMSD, root mean square deviation; TEGDMA, triethylene glycol dimethacrylate; UDMA, urethane dimethacrylate.

The AMPs pardaxin, tachystatin, and thermolysin emerged as top candidates based on their superior docking scores, binding affinities, and interaction metrics across various dental resin monomers. Pardaxin consistently demonstrated strong binding interactions with Bis‐GMA, EBPADMA, and UDMA, marked by favorable HADDOCK scores, low RMSD values, and high buried surface areas, indicating stable and extensive surface interactions. Similarly, tachystatin exhibited robust binding to Bis‐GMA, EBPADMA, and HEMA, with high cluster sizes and compact conformational stability. Thermolysin also displayed strong binding affinity, particularly with Bis‐GMA and EBPADMA, supporting its potential for multiresin applications. These findings suggest these peptides possess versatile interaction capabilities with typical dental composite monomers.

**Figure 2:**
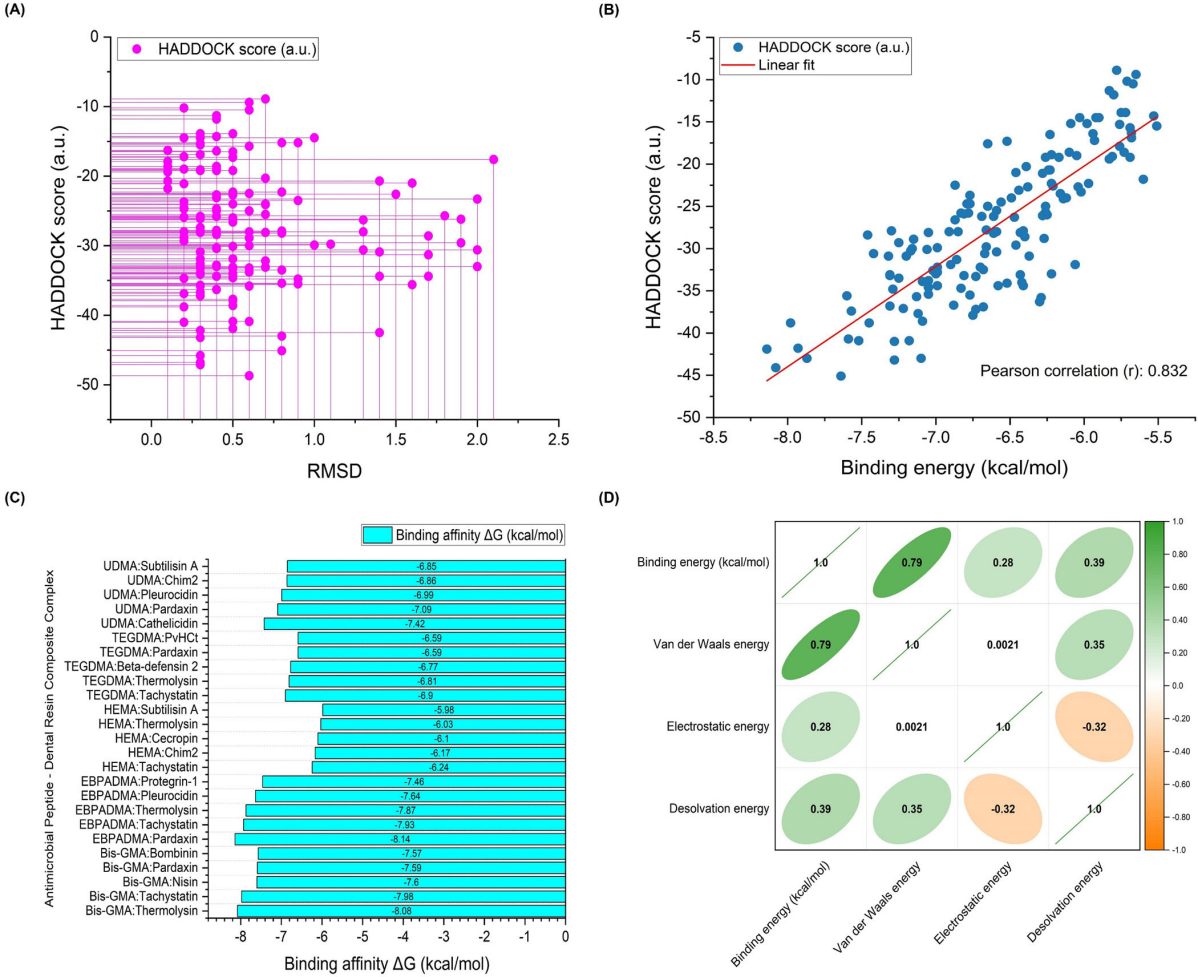
Comprehensive analysis of molecular docking results between antimicrobial peptides (AMPs) and dental resin composite components. (A) Distribution of HADDOCK scores versus root mean square deviation (RMSD) values for all docking poses. Each magenta point represents an individual docking pose. Lower HADDOCK scores indicate more favorable binding, while low RMSD values suggest higher structural convergence and pose stability. The wide range of RMSD reflects the structural variability across AMP–resin complexes. (B) Scatter plot showing a strong positive correlation between HADDOCK scores (a.u.) and binding energy values (kcal/mol) from AutoDock simulations. The Pearson correlation coefficient (*r* = 0.832) indicates that both scoring approaches yield consistent predictions regarding peptide–resin binding affinity. (C) Bar chart summarizing the most stable AMP–resin composite complexes with the lowest calculated binding energies. Each entry reflects the most favorable AMP–resin interaction for a given resin composite, suggesting potential candidates for future formulation development. (D) Correlation matrix of molecular interaction energy components. A strong positive correlation was found between binding energy and van der Waals energy (*r* = 0.79), indicating that these interactions play a key role in stabilizing the complexes. Desolvation energy showed a moderate positive correlation (*r* = 0.39), suggesting that solvent removal enhances binding. Electrostatic energy exhibited a weaker correlation (*r* = 0.28), indicating a smaller contribution to overall stability. Bis‐GMA, bisphenol A glycidyl methacrylate; EBPADMA, ethoxylated bisphenol A dimethacrylate; HADDOCK, High Ambiguity‐Driven protein–protein DOCKing; HEMA, 2‐hydroxyethyl methacrylate; TEGDMA, triethylene glycol dimethacrylate; UDMA, urethane dimethacrylate.

The molecular docking simulations produced AMP and dental resin composite combinations with most RMSD values below 2.0 Å, which displayed excellent structural alignment. In comparison, nearly 86.67% of the combinations had RMSD values below 1.0 Å, demonstrating substantial structural similarity between predicted and optimal conformations. The plot in Figure 2A illustrates how the HADDOCK score connects to RMSD measurements, representing the structural deviations between the predicted and optimal AMP–resin complex conformations. The plot demonstrates the relationship between HADDOCK score changes, which indicate variations in binding affinity and structural similarity quantified by RMSD values. Reduced RMSD values show that the predicted conformations are more closely matched to their optimal counterparts, demonstrating better accuracy in forecasting binding interactions (Dermawan et al. 2019; Kufareva and Abagyan 2012). The observed relationship confirmed that docking simulations reliably predicted successful AMP–resin binding interactions. Figure 2B illustrates a strong Pearson's correlation coefficient of 0.832 between HADDOCK score and binding affinity. The correlation coefficient confirmed a strong positive relationship between the two parameters. A high coefficient value close to 1 indicates that improved HADDOCK scores correlate directly with a higher binding affinity. When the HADDOCK score diminishes, it leads to weaker binding affinity. This meaningful correlation demonstrated that the HADDOCK scoring system accurately predicted the binding affinities of AMP–resin complexes and proved its reliability. The observed consistency between the computational predictions and experimental outcomes strengthens the credibility of the molecular docking approach used in this study.

The experimental data revealed substantial binding affinity variations among the different AMP–resin combinations. The measurement of the binding energy provided crucial information about the thermodynamic stability of the AMP–resin interactions. Figure 2C displays the best AMP–resin combinations for every dental resin variant by showing the lowest binding energy measurements, which showed a higher binding affinity. Figure 2D displays a correlation matrix that maps the interactions between the binding energy and various energy components, such as van der Waals, electrostatic, and desolvation energy. The matrix is an advanced analytical tool that provides detailed insights into how different energy factors interact within the AMP–resin complexes. Each cell in the correlation matrix displays a coefficient that measures the strength and direction of the connection between the binding energy and its respective energy component. The coefficients have values between −1 and 1, with positive numbers showing positive correlations, negative numbers indicating negative correlations, and values close to zero demonstrating little to no correlation between variables. A coefficient value of 1 denotes a perfect positive correlation, meaning that any increase in one variable leads to an identical increase in the other variable. A coefficient of −1 demonstrates an ideal negative correlation because decreases in one variable compensate for increases in the other variable. When the correlation coefficient equals 0, the variables show no linear relationship with each other. Analysis of the correlation coefficients revealed interesting dynamics between AMP and resin interactions. The van der Waals energy demonstrated a strong positive correlation coefficient of 0.79. These data indicate that van der Waals forces are fundamental to the stability of molecular complexes while enhancing their stability and binding affinity characteristics. Molecules that get close to each other without establishing chemical bonds highlight the significance of van der Waals interactions, which emerge from electron distribution fluctuations within the molecules (Hermann et al. 2017; Al‐Hamdani and Tkatchenko 2019). These interactions provide essential stabilization, preserving the AMP–resin complexes' structural integrity and functional effectiveness. The proper alignment and orientation of AMP molecules on resin surfaces enables enhanced antibacterial properties when AMPs are used in dental materials.

The positive correlation coefficient of electrostatic energy (*r* = 0.28) showed that electrostatic interactions helped stabilize the complexes, but to a lesser extent than van der Waals interactions. The interaction of electrostatic forces occurs through attraction or repulsion between charged particles, such as ions or polar molecules, depending on the molecular charge distribution (Hattori et al. 2013; Hurd 1999). Electrostatic forces within AMP–resin complexes emerge from interactions between the charged or polar functional groups on the AMP and resin surfaces. The moderately positive correlation coefficient of the desolvation energy (*r* = 0.39) demonstrated that desolvation plays a critical role in improving the stability of the AMP–resin complex. The desolvation process requires the removal of solvent molecules from the AMP–resin interface, which leads to an energy penalty (Cui et al. 2021). Correlation analysis demonstrated that the desolvation energy significantly affected the overall binding strength of the complexes. The desolvation process enhances bond formation between AMP and resin by removing solvent molecules from their interface, resulting in better complex stability and affinity properties (Hussain and Maktedar 2023; C. Yuan et al. 2023).

### 3.2 Molecular Docking Simulations of AMPs and Surface Protein Adhesin

**Figure 3:**
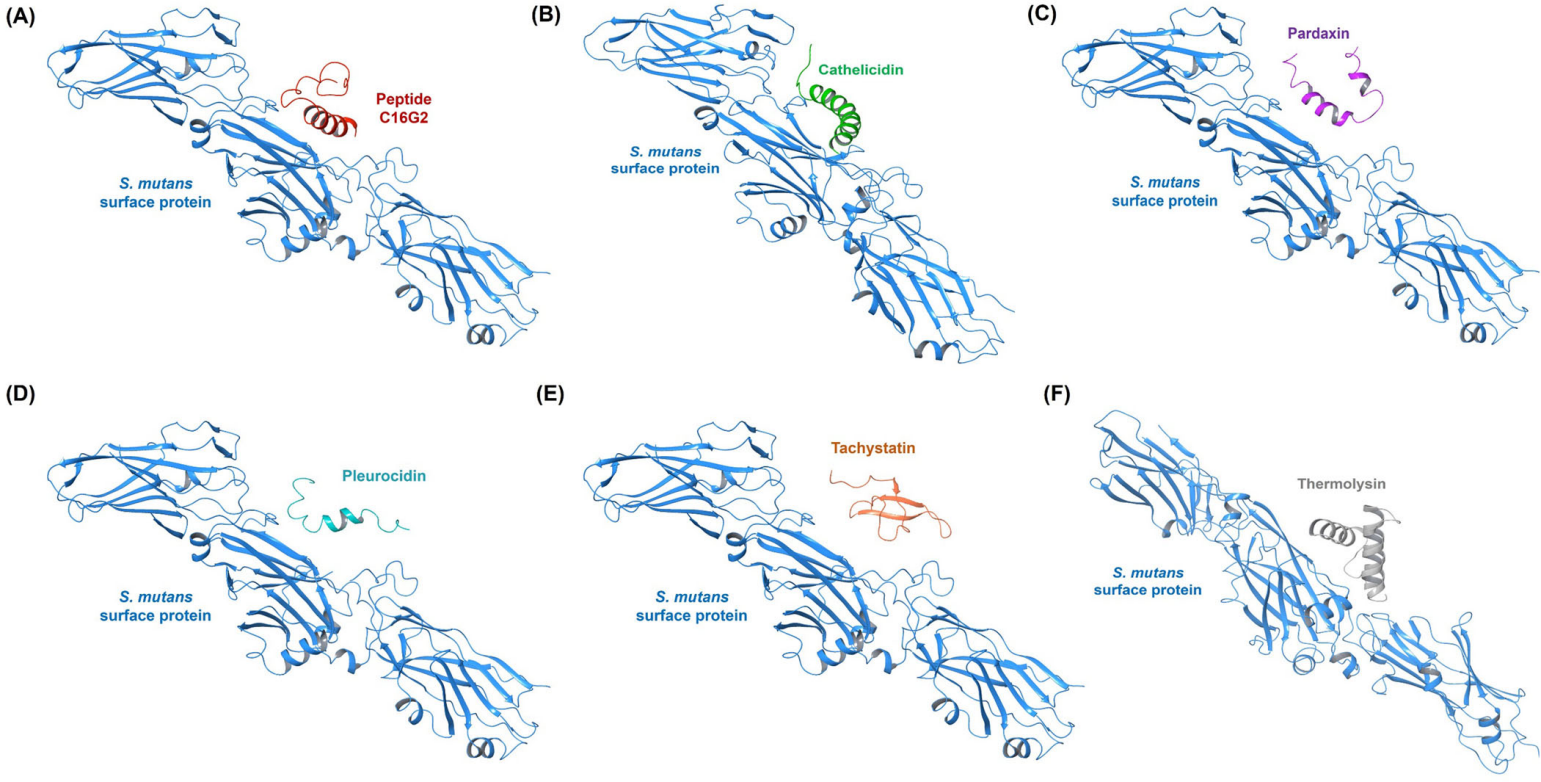
Visual representation of optimal binding orientations between antimicrobial peptides (AMPs) and *Streptococcus mutans* surface protein adhesin based on molecular docking simulations. (A) Reference complex of the C16G2 peptide (red) bound to the *S. mutans* adhesin (blue ribbon), serving as the standard comparator. (B) Cathelicidin (green) displays deep groove interaction with the protein surface. (C) Pardaxin (magenta) binds via α‐helical insertion close to the binding cleft. (D) Pleurocidin (teal) shows peripheral association along the β‐sheet domain. (E) Tachystatin (orange) exhibits compact, loop‐based binding on the outer surface. (F) Thermolysin (gray) forms stable contacts through its helical region near the active site.

Building on the promising findings with dental resin composites, we further assessed the binding interactions of the top five AMPs, including cathelicidin, pardaxin, pleurocidin, tachystatin, and thermolysin, with surface protein adhesin. This study evaluated these AMPs to understand their binding potential and interaction patterns with essential biological proteins and determine their functional applicability (Figure 3). Molecular docking simulations of AMP–surface protein adhesin complexes revealed vital information regarding their binding affinities and interaction dynamics. The results of the simulations are detailed in Table 4, highlighting important parameters, including the HADDOCK score, binding energy, van der Waals energy, electrostatic energy, desolvation energy, and RMSD. The complex between pardaxin and surface protein adhesin displayed the strongest binding interactions with a HADDOCK score of −126.7 ± 7.0 a.u. and a binding energy of −11.5 kcal/mol. The strong affinity between pardaxin and surface protein adhesin demonstrated that this peptide could effectively boost antimicrobial activity in dental resin composites. Favorable van der Waals interactions (−38.2 ± 7.1) maintain the pardaxin complex structure and contribute to its stability. The large magnitude of negative electrostatic energy (−156.1 ± 16.9) demonstrates that charged groups in the peptide and protein establish attractive forces.

**Table 4:** Molecular docking results of the top five dental resin composite‐antimicrobial peptide (AMP) complexes.

Surface protein adhesin: AMP complex	HADDOCK score (a.u.)	Binding energy (kcal/mol)	van der Waals energy	Electrostatic energy	Desolvation energy	RMSD
*Standard inhibitor*						
Surface protein adhesin:peptide C16G2	−70.3 ± 1.6	−7.7	−34.5 ± 6.0	−262.6 ± 25.8	7.7 ± 2.7	1.2 ± 0.5
*AMPs complexes*						
Surface protein adhesin:cathelicidin	−118.6 ± 2.0	−9.1	−0.7 ± 7.1	−687.4 ± 30.6	10.5 ± 3.5	0.7 ± 0.6
Surface protein adhesin:pardaxin	−126.7 ± 7.0	−11.5	−38.2 ± 7.1	−156.1 ± 16.9	−14.8 ± 1.4	1.2 ± 0.2
Surface protein adhesin:pleurocidin	−97.0 ± 2.5	−10.0	−37.1 ± 5.1	−248.0 ± 9.3	−12.8 ± 3.1	1.1 ± 0.9
Surface protein adhesin:tachystatin	−98.5 ± 17.3	−9.6	−43.5 ± 10.6	−223.7 ± 12.4	−5.2 ± 5.0	1.0 ± 0.5
Surface protein adhesin:thermolysin	−81.4 ± 2.3	−10.4	−44.9 ± 3.0	−198.8 ± 21.3	−5.4 ± 1.7	1.3 ± 0.1

Abbreviations: HADDOCK, High Ambiguity‐Driven protein–protein DOCKing; RMSD, root mean square deviation.

The AMP cathelicidin demonstrated strong binding affinity with a HADDOCK score of −118.6 ± 2.0 a.u. with a binding energy of −9.1 kcal/mol. The binding stability of the cathelicidin complex depends mainly on electrostatic interactions, which are essential for its attachment to surface protein adhesin. HADDOCK scores showed pleurocidin and tachystatin complexes to have moderate binding affinities at −97.0 ± 2.5 and −98.5 ± 17.3 a.u. The binding energies of −10.0 and −9.6 kcal/mol show these AMPs might remain effective despite their scores, indicating weaker interactions than those of pardaxin and cathelicidin. Thermolysin demonstrated a reasonable binding energy of −10.4 kcal/mol yet achieved the lowest HADDOCK score of −81.4 ± 2.3 a.u. Thermolysin showed the lowest HADDOCK score among the AMPs, implying reduced antimicrobial activity effectiveness in dental resin composites.

Table 5 presents comprehensive data concerning the intermolecular contacts (ICs) and noninteracting surface (NIS) areas of surface protein adhesin complexes with the standard antagonist peptide C16G2 and AMPs to explore these molecular interactions further. The information gathered detailed the specific IC types and quantities, including charged–charged, charged–polar, charged–apolar, polar–polar, polar–apolar, and apolar–apolar interactions. Cathelicidin achieved the most ICs, amounting to 52, with interaction counts of 12 charged–charged, 8 charged–polar, and 18 charged–apolar. The strong interaction network between cathelicidin and the surface protein adhesin increases binding affinity and stability, as suggested by the high total number of ICs. Pardaxin displayed 59 ICs, as determined by its three charged–charged and 24 notable polar–apolar interactions. The numerous polar–apolar interactions in pardaxin enable it to bind effectively to adhesin's hydrophobic areas, enhancing its binding characteristics. Both pleurocidin and tachystatin showed similar quantities of ICs, where pleurocidin had 48 ICs and tachystatin had 46 ICs. The interaction profile of pleurocidin demonstrates a balance through its seven charged–charged contacts and 10 apolar–apolar interactions, which lead to effective binding capabilities. The binding ability of tachystatin to surface protein adhesin is potentially enhanced by its 5 charged–charged and 8 polar–apolar interactions.

**Table 5:** Intermolecular contacts and noninteracting surface areas of surface protein adhesin complexes with standard inhibitors and antimicrobial peptides (AMPs).

Surface protein adhesin: AMP complex	ICs charged–charged	ICs charged–polar	ICs charged–apolar	ICs polar–polar	ICs polar–apolar	ICs apolar–apolar	NIS charged	NIS apolar
*Standard inhibitor*								
Surface protein adhesin:peptide C16G2	6	9	12	4	7	3	26.55	38.14
*AMPs complexes*								
Surface protein adhesin:cathelicidin	12	8	18	1	6	7	27.95	37.95
Surface protein adhesin:pardaxin	3	3	15	4	24	13	23.96	40.34
Surface protein adhesin:pleurocidin	7	5	18	0	10	10	24.81	38.73
Surface protein adhesin:tachystatin	5	8	15	3	8	25	23.83	38.79
Surface protein adhesin:thermolysin	7	3	20	0	11	20	25.67	38.39

Abbreviations: ICs, number of intermolecular contacts; NIS, noninteracting surface.

Thermolysin's peptide demonstrated the most significant interaction contacts, totaling 61, including 7 charged–charged, 3 charged–polar, and 20 charged–apolar interactions among all tested peptides. The substantial presence of charged–apolar contacts shows thermolysin's strong affinity toward particular binding sites on adhesin. Still, its interaction versatility appears limited due to the absence of polar–polar contacts. The standard inhibitor peptide C16G2 demonstrated 41 total ICs, equal distribution across all interaction categories. The molecule displayed 6 charged, 9 charged, and 12 charged–apolar interactions, which allowed it to interact with various features of the surface protein adhesin. The evaluation of NIS areas showed that pardaxin exhibited a minimal charged NIS measurement of 23.96 Å^2^ among AMPs, indicating superior packing efficiency and surface area usage for binding operations. Cathelicidin exhibited a slightly larger charged NIS (27.95 Å^2^), showing reduced efficiency in charged interactions compared with pardaxin. Thermolysin possesses the most considerable apolar NIS value (20.39 Å^2^) among the complexes, which could affect its binding performance and interaction characteristics.

### 3.3 MD Simulation

**Figure 4:**
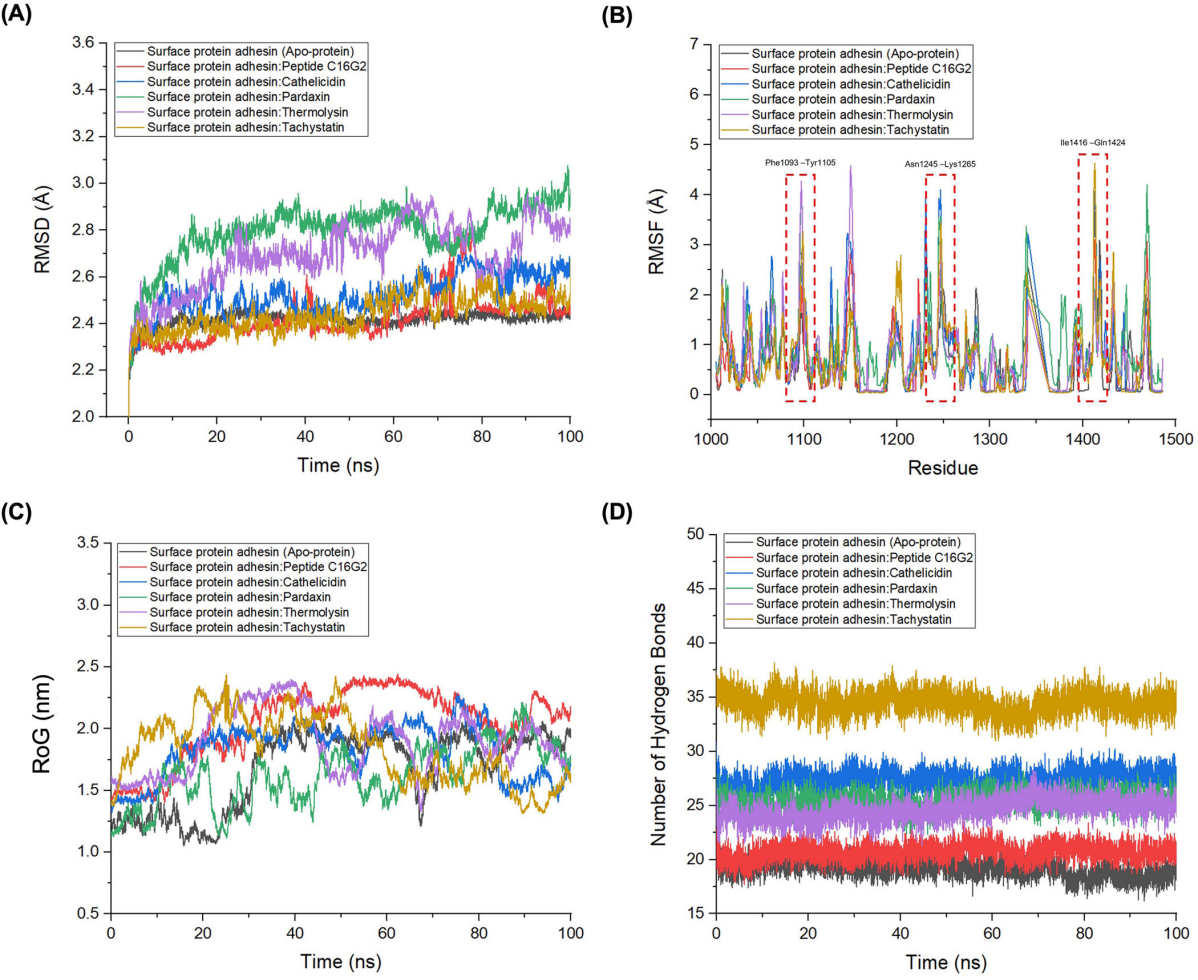
Molecular dynamics (MD) simulation analysis of the complexes between surface protein adhesins and AMPs focused on several critical parameters: (A) root mean square deviation (RMSD) to evaluate structural stability, (B) root mean square fluctuation (RMSF) to assess residue flexibility, (C) radius of gyration (RoG) to represent overall structural compactness, and (D) number of hydrogen bonds to examine intermolecular interactions. AMP, antimicrobial peptide.

MD simulation findings extensively explain how protein‐protein complexes emerge between surface protein adhesins and AMPs. The surface protein adhesin exhibited a stable structure during the 100 ns simulation period, as evidenced by RMSD values between 2.392 and 2.796 Å, which showed no significant variations (Figure 4A). The simulation results showed that the binding interactions between the surface protein adhesin and the standard inhibitor and AMPs did not change during the entire simulation timeframe. RMSD values demonstrated significant disparities between different protein complexes when assessing the extent of structural deviation from the initial conformations. The average RMSD for the surface protein adhesin–peptide C16G2 complex was slightly higher (2.424 Å) than that for the apo‐protein surface protein adhesin (RMSD = 2.392 Å). The analysis indicated that binding to the standard inhibitor resulted in moderate structural flexibility enhancement, suggesting that conformational shifts are necessary for proper binding. In addition, the AMP complexes cathelicidin, pardaxin, tachystatin, and thermolysin presented elevated average RMSD values between 2.439 and 2.796 Å when measured against the apo‐protein. AMP binding generates potential differences in the dynamic behavior and conformational changes in proteins.

RMSF analysis during MD simulations assessed the flexibility of individual amino acid residues in the surface protein adhesin. Protein regions exhibited moderate flexibility according to RMSF measurements, ranging between 0.628 and 0.931 Å. Through analysis, we gained insights into how specific residues move and how flexible they are, which helps us understand their roles in protein structures (Craveur et al. 2015; Saini et al. 2024b). When the leading AMP complex binds to the surface protein adhesin, it disrupts key hydrogen bonds in the regions between residues Phe1093 and Tyr1105, Asn1245–Lys1265, and Ile1416–Gln1424 (Figure 4B), which are essential for the active binding site of the protein. The dissociation of hydrogen bonds in essential regions increases residue fluctuations compared with the unbound protein state. Proteins exhibit increased movement and adaptability when AMPs attach to them. The dynamic process of interaction breaking and reforming results in the alteration of the conformational landscape of the protein. The dynamic increase in flexibility from top‐performing AMPs showed similarities to the behavior of peptide C16G2, which serves as a standard inhibitor, suggesting a comparable mechanism of action for AMPs as effective protein inhibitors. RMSF analysis showed that AMP attachment to surface protein adhesin leads to functional changes through increased flexibility in crucial regions, enabling peptide inhibition. Understanding these dynamics will enable the logical development of AMPs with improved binding strength and precision. Peptides that target regions to enhance flexibility and break crucial interactions offer a method for controlling protein function, which opens new therapeutic possibilities.

MD simulations have yielded valuable data on the compactness or expansion of protein structures using RoG (Sanusi and Lobb 2022). This study evaluated the RoG values of surface protein adhesin complexes to understand the effect of different AMPs on the structural integrity and compactness of these proteins. Protein adhesin complexes displayed average RoG values between 1.621 and 2.048 nm when bound to other peptides, showing how protein compactness varied across these complexes (Figure 4C). The standard antagonist complex with peptide C16G2 registered an RoG value of 1.824 nm, which established a baseline for the compactness of the protein upon inhibitor binding. The AMP‐bound complexes demonstrated slightly elevated RoG values, extending from 1.839 to 2.048 nm. Higher RoG values indicate that AMP‐bound protein structures are expanded or maintained less compact than proteins bound to the standard antagonist. Different binding modes and interaction patterns of AMPs with surface protein adhesin cause variations in compactness.

The number of hydrogen bonds between the surface protein adhesin and the interacting peptides was a key metric for measuring the interaction strength and specificity (Dermawan et al. 2021; Dermawan and Alotaiq 2025b). Hydrogen bonds were calculated using the standard donor–acceptor criteria: a maximum donor–acceptor distance of 3.5 Å and a hydrogen–donor–acceptor angle cutoff of 30°. Protein–peptide complex stability relies heavily on hydrogen bonds, which enhance binding affinity. Surface protein adhesin: The formation of 22 hydrogen bonds by the peptide C16G2 complex provided an initial reference point for comparison. The hydrogen bond count in the AMP complexes varied between 25 and 37, which was higher than that of the other complexes. Notably, the surface protein adhesin:tachystatin complex demonstrated the highest hydrogen bond total, reaching 37 bonds. This was followed closely by the surface protein adhesin:cathelicidin complex established 28 hydrogen bonds, while the surface protein adhesin:pardaxin complex established 26 hydrogen bonds, as shown in Figure 4D. The elevated number of hydrogen bonds in these AMP complexes indicates more substantial and more specific AMP–surface protein adhesin interactions.

### 3.4 MM/PBSA Calculations

MM/PBSA calculations were used to determine the binding free energy of the surface protein adhesin complexes in combination with the most effective AMPs and the reference inhibitor peptide C16G2. The binding free energy serves as a key metric for analyzing the stability and affinity of these complexes and demonstrates the thermodynamic favorability of their interactions. Through three separate computational runs, the surface protein adhesin–peptide C16G2 complex demonstrated an average Δ*G*_binding_ of −33.34 kcal/mol based on individual results of −33.77, −33.70, and −32.57 kcal/mol. The surface protein adhesin binds stably to peptide C16G2, as shown by the moderate average binding free energy, but its less negative value indicates weaker interaction strength compared with AMPs (Table 6).

**Table 6:** The average binding free energy (Δ*G*_binding_) for the surface protein adhesin complexes was reported as a standard deviation in kcal/mol units, as determined by MM/PBSA calculations.

Complex	MM/PBSA calculation results Δ*G*_binding_ (kcal/mol)			Average (kcal/mol)
	I	II	III	
*Standard inhibitor*				
Surface protein adhesin:peptide C16G2	−33.77	−33.70	−32.57	−33.34
*Antimicrobial peptides (AMPs) complexes*				
Surface protein adhesin:cathelicidin	−43.69	−43.65	−42.67	−43.33
Surface protein adhesin:pardaxin	−38.42	−39.13	−39.03	−38.86
Surface protein adhesin:pleurocidin	−46.72	−46.65	−46.69	−46.68
Surface protein adhesin:tachystatin	−62.10	−62.03	−61.98	−62.03
Surface protein adhesin:thermolysin	−43.09	−42.50	−43.72	−43.10

Abbreviation: MM/PBSA, molecular mechanics/Poisson–Boltzmann surface area.

AMP complexes showed various binding free energies, demonstrating different interaction strengths with surface protein adhesin. For the surface protein adhesin–cathelicidin complex, the average Δ*G*_binding_ came to −43.33 kcal/mol while the individual measurements were −43.69, −43.65, and −42.67 kcal/mol. The interaction between cathelicidin and the surface protein adhesin appears much stronger than that of peptide C16G2 because cathelicidin establishes a stable and functional complex with the surface protein adhesin. The binding energy for the surface protein adhesin–pardaxin complex averaged −38.86 kcal/mol, with values of −38.42, −39.13, and −39.03 kcal/mol observed individually. Pardaxin maintains a favorable binding interaction despite having a lower negative average binding free energy than cathelicidin, demonstrating its effective binding to the surface protein adhesin. The surface protein adhesin–pleurocidin complex demonstrated a strong average Δ*G*_binding_ of −46.68 kcal/mol while individual measurements recorded values of −46.72, −46.65, and −46.69 kcal/mol. The strong complex formation between pleurocidin and surface protein adhesin demonstrates its potential as an effective antimicrobial agent. The surface protein adhesin–tachystatin complex showed the strongest binding interaction with an average Δ*G*_binding_ of −62.03 kcal/mol, which was calculated from values of −62.10, −62.03, and −61.98 kcal/mol. The tachystatin molecule demonstrates a very high binding affinity toward the surface protein adhesin because its Δ*G*_binding_ value is significantly lower than others, forming the most stable complex among all AMPs tested. The average binding free energy for the surface protein adhesin–thermolysin complex reached −43.10 kcal/mol according to individual data points of −43.09, −42.50, and −43.72 kcal/mol. The binding strength is moderate but does not surpass the performance of the best AMPs.

## 4 Discussion

According to the simulations, different AMPs show unique interaction patterns with dental resin composites, which are mainly defined by hydrophobic interactions and the formation of hydrogen bonds along with salt bridges. The stability and performance of the AMP–resin composite depend on these interactions. Researchers have identified several AMP–resin pairs demonstrating high affinity and stability during their interactions. The Bis‐GMA and thermolysin interactions strongly bind by forming multiple hydrophobic interactions and a single salt bridge. Pardaxin and cathelicidin formed strong specific bonds with EBPADMA through hydrophobic interactions and hydrogen bonds, demonstrating their potential effectiveness in this dental resin composite. The effectiveness of pleurocidin and tachystatin in resin composite performance shows promising binding interactions, illustrating the multiple mechanisms these AMPs utilize to improve performance. While previous works have explored antibacterial coatings or AMP‐functionalized hydrogels, studies integrating AMPs directly into dental resins are still emerging. Our results expand on recent experimental efforts where AMP–resin hybrids demonstrated improved antimicrobial efficacy, although long‐term material behavior remains under‐investigated. Beyond microbial inhibition, AMP binding may also influence the mechanical properties of resin materials. Stable peptide incorporation, particularly via hydrophobic and hydrogen bonding, can alter polymer network formation and potentially improve resistance to biofilm penetration. However, excessive peptide content or leaching could compromise material stiffness or longevity, indicating the need for fine‐tuned AMP loading.

The study findings confirm earlier research highlighting hydrophobic and electrostatic interactions as key factors for peptide–resin composite stability, demonstrating their significant impact on dental application performance (Moussa et al. 2019; Delaviz et al. 2014). Our study showed that the AMPs tested interacted with resin composites differently, with some AMPs forming stronger and more extensive bonds. The peptide tachystatin formed numerous hydrogen bonds and hydrophobic interactions with HEMA, pointing to a stable and specific binding interface. The current study supports previous findings that peptides containing numerous hydrogen bonds demonstrate stronger binding affinities and improved stability during biomaterial interactions (Zhou et al. 2024; Gan et al. 2021).

The binding capacities of top AMPs, including pardaxin, cathelicidin, pleurocidin, tachystatin, and thermolysin, were examined against the surface protein adhesin following AMP–resin interaction studies. This assessment focused on determining the extensive potential of these AMPs by exploring their interactions with biologically significant proteins. Docking simulations demonstrated that all five AMPs bound strongly to the surface protein adhesin. Still, pardaxin showed the strongest binding performance because of considerable van der Waals and electrostatic interactions, which enhanced its stability. Cathelicidin and tachystatin demonstrated robust binding through substantial electrostatic interactions, whereas pleurocidin and thermolysin showed strong but comparatively less favorable binding capabilities. Our findings align with earlier studies on peptide–protein interactions, demonstrating that van der Waals forces and electrostatic interactions play crucial roles in binding affinity and stability. Research has shown that peptide–protein complexes achieve greater stability and efficacy through van der Waals and electrostatic interactions. This supports our findings regarding AMP–surface protein adhesin interactions (Zhu et al. 2021; Hosseinpour et al. 2020). A detailed study of ICs clarified how AMPs interact with the surface protein adhesin. The peptide pardaxin demonstrated strong binding affinity through several interactions, including charged, polar, and apolar bonds. A previous study established the importance of multiple noncovalent interactions in peptide–protein complex stabilization and emphasized that diverse interaction types improve binding stability and effectiveness (Adhav and Saikrishnan 2023). MD simulations revealed the dynamic behavior of AMP–surface protein adhesin complexes. AMP binding causes specific regions of the surface protein adhesin to become more flexible while maintaining its overall stable conformation, indicating the need for conformational changes to enable effective binding. Scientific research has shown that when peptides bind to target proteins, they cause structural changes that increase flexibility and functional state alterations. Studies demonstrate that these conformational changes provide essential insights into peptide binding functions (Gupta et al. 2022; Al Qaraghuli et al. 2020).

Clinically, AMP‐functionalized dental resins have been explored in a few in vivo and ex vivo studies, where embedded peptides showed promising short‐term antibacterial effects and acceptable biocompatibility (Meneses et al. 2020; Algarni 2024). However, major barriers remain, particularly related to AMP leaching, degradation, and long‐term retention of activity. AMPs may undergo enzymatic degradation or diffusion from the resin matrix, potentially reducing effectiveness or triggering adverse host responses, such as cytotoxicity or immunogenicity (Canè et al. 2024; Eckert 2011). Thus, whether AMP‐functionalized resins can retain their antimicrobial activity over extended clinical use remains a critical question. Future development must emphasize strategies for covalent immobilization or controlled‐release formulations to ensure durable peptide activity while preserving resin integrity.

## 5 Limitations and Clinical Considerations

Molecular docking simulations reveal essential details regarding how AMPs interact with dental resin composites, offering a foundation for the rational design of antimicrobial dental materials. However, this study has several limitations that must be considered when interpreting the findings. First, no experimental validation (e.g., minimum inhibitory concentration testing, cytotoxicity assays, or mechanical property evaluation) was performed to confirm the biological activity, safety, or functional impact of AMP–resin integration. Second, only one bacterial target, *S. mutans*, was modeled, which limits the generalizability of the results to broader oral microbiota relevant to caries or periodontal disease. Third, docking simulations were based on static molecular structures, which do not fully capture the dynamic conformational changes that occur in biological systems. This may lead to overestimation of binding affinities and interaction specificity. Molecular interactions in vivo are influenced by factors, such as temperature fluctuations, solvent effects, protein flexibility, and enzymatic degradation, none of which are adequately represented in rigid‐body docking. As prior research has shown, static models can oversimplify complex molecular interactions and reduce predictive reliability (Pantsar and Poso 2018; Meng et al. 2011). Moreover, the exclusive use of a single docking method may introduce algorithm‐specific bias; future work should incorporate multiple docking algorithms and MD simulations for more comprehensive modeling. While our in silico findings provide meaningful insight into AMP–resin interactions, they should be considered as hypothesis‐generating rather than conclusive and must be followed by empirical validation through biophysical techniques, such as surface plasmon resonance (SPR) and isothermal titration calorimetry (ITC). SPR enables real‐time, label‐free measurement of molecular binding kinetics (association and dissociation rates) between AMPs and target surfaces, helping to quantify interaction strength and specificity (Sparks et al. 2019). ITC, on the other hand, measures the heat changes during binding events, providing direct information on binding affinity (Kd), enthalpy (Δ*H*), and stoichiometry of interactions, making it highly suitable for evaluating AMP–resin or AMP–protein interactions (Damian 2013). Additional clinical considerations include the potential for AMP leaching from resin matrices, degradation over time, cytotoxicity to oral tissues, and the risk of immunogenicity, which could limit long‐term clinical application. The long‐term activity of embedded AMPs remains uncertain and must be evaluated under simulated oral conditions, including exposure to mechanical wear, salivary enzymes, and fluctuating pH. Importantly, the potential for bacterial resistance to AMPs, similar to antibiotic resistance, must be considered and monitored. Finally, cost‐effectiveness and manufacturing feasibility are key to transitioning AMP‐functionalized resins from bench to clinic. Bridging the gap between computational predictions and real‐world applications will require a multidisciplinary strategy combining bioinformatics, materials science, microbiology, and clinical trials.

## 6 Conclusion and Future Works

Our research demonstrates the potential benefits of incorporating AMPs, pardaxin, cathelicidin, tachystatin, and thermolysin into dental resin composites to improve their ability to fight bacteria. Pardaxin and cathelicidin display broad‐spectrum effectiveness against multiple pathogenic bacteria, whereas tachystatin and thermolysin provide distinctive mechanisms that will enhance biofilm management and reduce bacterial resistance. Molecular docking simulations revealed important information regarding how these AMPs interact with dental resin materials and suggested that they could significantly improve the antimicrobial effectiveness of dental composites. These results highlight the need for additional validation studies, both in vitro and in vivo, to assess the safety profile and long‐term stability of dental materials enhanced with AMPs. Subsequent research needs to focus on the identified limitations of this study, which include the dynamic interactions within biological systems and the effect of biological fluids on AMP functionality. Research into different AMP concentrations, release kinetics, and clinical resistance development must be conducted to enable these findings to become practical applications. The use of multiple docking methods together with various experimental approaches will improve our prediction accuracy and expand our knowledge of AMP–resin interactions. The cost‐efficiency of adding AMPs to dental composites requires evaluation in future research, while their use in other biomedical applications demands exploration owing to their necessary antimicrobial properties.

## Author Contributions

**Ravinder S. Saini:** conceptualization, methodology, writing – original draft, project administration, supervision. **Abdulkhaliq Ali F. Alshadidi:** writing – review and editing, visualization, software. **Doni Dermawan:** formal analysis, data curation, resources, investigation, writing – review and editing. **Lujain Ibrahim N. Aldosari:** data curation, formal analysis, writing – review and editing. **Rayan Ibrahim H. Binduhayyim:** investigation, resources, writing – review and editing. **Rajesh Vyas:** data curation, formal analysis, writing – review and editing. **Sunil Kumar Vaddamanu:** resources, investigation, writing – review and editing. **Mohamed Saheer Kuruniyan:** writing – original draft, software, visualization. **Artak Heboyan:** supervision, project administration, writing – review and editing, formal analysis, validation, visualization.

## Ethics Statement

The authors have nothing to report.

## Conflicts of Interest

The authors declare no conflicts of interest.

## Supporting information

Supplementary Data 1 ‐ Antimicrobial Peptides Dataset.

Supplementary Data 2 ‐ Molecular Docking Results.

## Data Availability

The data that support the findings of this study are available on request from the corresponding author. The data are not publicly available due to privacy or ethical restrictions.

## References

[mbo370116-bib-0001] Adhav, V. A. , and K. Saikrishnan . 2023. “The Realm of Unconventional Noncovalent Interactions in Proteins: Their Significance in Structure and Function.” ACS Omega 8, no. 25: 22268–22284. 37396257 10.1021/acsomega.3c00205 PMC10308531

[mbo370116-bib-0002] Algarni, A. A. 2024. “Antibacterial Agents for Composite Resin Restorative Materials: Current Knowledge and Future Prospects.” Cureus 16, no. 3: e57212. 38681374 10.7759/cureus.57212 PMC11056222

[mbo370116-bib-0003] Al‐Hamdani, Y. S. , and A. Tkatchenko . 2019. “Understanding Non‐Covalent Interactions in Larger Molecular Complexes From First Principles.” Journal of Chemical Physics 150, no. 1: 010901. 30621423 10.1063/1.5075487 PMC6910608

[mbo370116-bib-0004] Alotaiq, N. , D. Dermawan , and N. E. Elwali . 2024. “Leveraging Therapeutic Proteins and Peptides From Lumbricus Earthworms: Targeting SOCS2 E3 Ligase for Cardiovascular Therapy Through Molecular Dynamics Simulations.” International Journal of Molecular Sciences 25, no. 19: 10818. 39409145 10.3390/ijms251910818 PMC11477351

[mbo370116-bib-0005] Al Qaraghuli, M. M. , K. Kubiak‐Ossowska , V. A. Ferro , and P. A. Mulheran . 2020. “Antibody–Protein Binding and Conformational Changes: Identifying Allosteric Signalling Pathways to Engineer a Better Effector Response.” Scientific Reports 10, no. 1: 13696. 32792612 10.1038/s41598-020-70680-0 PMC7426963

[mbo370116-bib-0006] Amos, S. B. T. A. , L. S. Vermeer , P. M. Ferguson , et al. 2016. “Antimicrobial Peptide Potency Is Facilitated by Greater Conformational Flexibility When Binding to Gram‐Negative Bacterial Inner Membranes.” Scientific Reports 6: 37639. 27874065 10.1038/srep37639 PMC5118786

[mbo370116-bib-0007] Bhunia, A. , P. N. Domadia , J. Torres , K. J. Hallock , A. Ramamoorthy , and S. Bhattacharjya . 2010. “NMR Structure of Pardaxin, a Pore‐Forming Antimicrobial Peptide, in Lipopolysaccharide Micelles.” Journal of Biological Chemistry 285, no. 6: 3883–3895. 19959835 10.1074/jbc.M109.065672 PMC2823531

[mbo370116-bib-0008] Canè, C. , L. Tammaro , A. Duilio , and A. Di Somma . 2024. “Investigation of the Mechanism of Action of AMPs From Amphibians to Identify Bacterial Protein Targets for Therapeutic Applications.” Antibiotics (USSR) 13, no. 11: 1076. 10.3390/antibiotics13111076 PMC11591259 39596769

[mbo370116-bib-0009] Comeau, P. A. , and T. L. Willett . 2020. “Triethyleneglycol Dimethacrylate Addition Improves the 3D‐Printability and Construct Properties of a GelMA‐nHA Composite System Towards Tissue Engineering Applications.” Materials Science & Engineering, C: Materials for Biological Applications 112: 110937. 32409083 10.1016/j.msec.2020.110937

[mbo370116-bib-0010] Comert, F. , A. Greenwood , J. Maramba , et al. 2019. “The Host‐Defense Peptide Piscidin P1 Reorganizes Lipid Domains in Membranes and Decreases Activation Energies in Mechanosensitive Ion Channels.” Journal of Biological Chemistry 294, no. 49: 18557–18570. 31619519 10.1074/jbc.RA119.010232 PMC6901303

[mbo370116-bib-0011] Craveur, P. , A. P. Joseph , J. Esque , et al. 2015. “Protein Flexibility in the Light of Structural Alphabets.” Frontiers in Molecular Biosciences 2: 1–20. 26075209 10.3389/fmolb.2015.00020 PMC4445325

[mbo370116-bib-0012] Crowley, P. J. , L. J. Brady , S. M. Michalek , and A. S. Bleiweis . 1999. “Virulence of a spaP Mutant of *Streptococcus mutans* in a Gnotobiotic Rat Model.” Infection and Immunity 67, no. 3: 1201–1206. 10024561 10.1128/iai.67.3.1201-1206.1999 PMC96447

[mbo370116-bib-0013] Cui, L. , H. Wang , S. Chen , et al. 2021. “The Interaction Energy Between Solvent Molecules and Graphene as an Effective Descriptor for Graphene Dispersion in Solvents.” Journal of Physical Chemistry C 125: 5167–5171.

[mbo370116-bib-0014] Damian, L. 2013. “Isothermal Titration Calorimetry for Studying Protein–Ligand Interactions.” Methods in Molecular Biology 1008: 103–118. 23729250 10.1007/978-1-62703-398-5_4

[mbo370116-bib-0015] Delaviz, Y. , Y. Finer , and J. P. Santerre . 2014. “Biodegradation of Resin Composites and Adhesives by Oral Bacteria and Saliva: A Rationale for New Material Designs That Consider the Clinical Environment and Treatment Challenges.” Dental Materials 30, no. 1: 16–32. 24113132 10.1016/j.dental.2013.08.201

[mbo370116-bib-0016] Dermawan, D. , and N. Alotaiq . 2025a. “Unveiling Pharmacological Mechanisms of *Bombyx mori* (Abresham), a Traditional Arabic Unani Medicine for Ischemic Heart Disease: An Integrative Molecular Simulation Study.” Pharmaceutics 17, no. 3: 295. 40142959 10.3390/pharmaceutics17030295 PMC11944354

[mbo370116-bib-0017] Dermawan, D. , and N. Alotaiq . 2025b. “Computational Analysis of Antimicrobial Peptides Targeting Key Receptors in Infection‐Related Cardiovascular Diseases: Molecular Docking and Dynamics Insights.” Scientific Reports 15, no. 1: 8896. 40087360 10.1038/s41598-025-93683-1 PMC11909139

[mbo370116-bib-0018] Dermawan, D. , B. A. Prabowo , and C. A. Rakhmadina . 2021. “In Silico Study of Medicinal Plants With Cyclodextrin Inclusion Complex as the Potential Inhibitors Against SARS‐CoV‐2 Main Protease (Mpro) and Spike (S) Receptor.” Informatics in Medicine Unlocked 25: 100645. 34189252 10.1016/j.imu.2021.100645 PMC8223117

[mbo370116-bib-0019] Dermawan, D. , R. Sumirtanurdin , and D. Dewantisari . 2019. “Molecular Dynamics Simulation Estrogen Receptor Alpha Againts Andrographolide as Anti Breast Cancer.” Indonesian Journal of Pharmaceutical Science and Technology 6, no. 2: 65–76.

[mbo370116-bib-0020] Dominguez, C. , R. Boelens , and A. M. J. J. Bonvin . 2003. “HADDOCK: A Protein−Protein Docking Approach Based on Biochemical or Biophysical Information.” Journal of the American Chemical Society 125, no. 7: 1731–1737. 12580598 10.1021/ja026939x

[mbo370116-bib-0021] Doni Dermawan, F. A. , N. E. Elwali , and N. Alotaiq . 2024. “Therapeutic Potential of Earthworm‐Derived Proteins: Targeting NEDD4 for Cardiovascular Disease Intervention.” Journal of Applied Pharmaceutical Science 15 1: 216–232.

[mbo370116-bib-0022] Eckert, R. 2011. “Road to Clinical Efficacy: Challenges and Novel Strategies for Antimicrobial Peptide Development.” Future Microbiology 6, no. 6: 635–651. 21707311 10.2217/fmb.11.27

[mbo370116-bib-0023] Fahrner, R. L. , T. Dieckmann , S. S. L. Harwig , R. I. Lehrer , D. Eisenberg , and J. Feigon . 1996. “Solution Structure of Protegrin‐1, a Broad‐Spectrum Antimicrobial Peptide From Porcine Leukocytes.” Chemistry & Biology 3, no. 7: 543–550. 8807886 10.1016/s1074-5521(96)90145-3

[mbo370116-bib-0024] Fiebig, D. , J. Storka , M. Roeder , et al. 2018. “Destructive Twisting of Neutral Metalloproteases: The Catalysis Mechanism of the Dispase Autolysis‐Inducing Protein From *Streptomyces mobaraensis* DSM 40487.” FEBS Journal 285, no. 22: 4246–4264. 30171661 10.1111/febs.14647

[mbo370116-bib-0025] Friedrich, C. L. , A. Rozek , A. Patrzykat , and R. E. W. Hancock . 2001. “Structure and Mechanism of Action of an Indolicidin Peptide Derivative With Improved Activity Against Gram‐Positive Bacteria.” Journal of Biological Chemistry 276, no. 26: 24015–24022. 11294848 10.1074/jbc.M009691200

[mbo370116-bib-0026] Fujitani, N. , S. Kawabata , T. Osaki , et al. 2002. “Structure of the Antimicrobial Peptide Tachystatin A.” Journal of Biological Chemistry 277, no. 26: 23651–23657. 11959852 10.1074/jbc.M111120200

[mbo370116-bib-0027] Gan, B. H. , J. Gaynord , S. M. Rowe , T. Deingruber , and D. R. Spring . 2021. “The Multifaceted Nature of Antimicrobial Peptides: Current Synthetic Chemistry Approaches and Future Directions.” Chemical Society Reviews 50, no. 13: 7820–7880. 34042120 10.1039/d0cs00729c PMC8689412

[mbo370116-bib-0028] Gesell, J. , M. Zasloff , and S. J. Opella . 1997. “Two‐Dimensional 1H NMR Experiments Show That the 23‐residue Magainin Antibiotic Peptide Is an α‐helix in Dodecylphosphocholine Micelles, Sodium Dodecylsulfate Micelles, and Trifluoroethanol/Water Solution.” Journal of Biomolecular NMR 9, no. 2: 127–135. 9090128 10.1023/a:1018698002314

[mbo370116-bib-0029] Grassmann, G. , M. Miotto , F. Desantis , et al. 2024. “Computational Approaches to Predict Protein–Protein Interactions in Crowded Cellular Environments.” Chemical Reviews 124, no. 7: 3932–3977. 38535831 10.1021/acs.chemrev.3c00550 PMC11009965

[mbo370116-bib-0030] Gupta, S. , N. Azadvari , and P. Hosseinzadeh . 2022. “Design of Protein Segments and Peptides for Binding to Protein Targets.” BioDesign Research 2022: 9783197. 37850124 10.34133/2022/9783197 PMC10521657

[mbo370116-bib-0031] Hattori, Y. , K. Kaneko , and T. Ohba . 2013. “5.02—Adsorption Properties.” In Comprehensive Inorganic Chemistry II, edited by J. Reedijk and K. Poeppelmeier , 2nd ed., 25–44. Elsevier.

[mbo370116-bib-0032] Heim, K. P. , P. J. Crowley , J. R. Long , S. Kailasan , R. McKenna , and L. J. Brady . 2014. “An Intramolecular Lock Facilitates Folding and Stabilizes the Tertiary Structure of *Streptococcus mutans* adhesin P1.” Proceedings of the National Academy of Sciences 111, no. 44: 15746–15751. 10.1073/pnas.1413018111 PMC4226092 25331888

[mbo370116-bib-0033] Hermann, J. , R. Distasio , and A. Tkatchenko . 2017. “First‐Principles Models for van der Waals Interactions in Molecules and Materials: Concepts, Theory, and Applications.” Chemical Reviews 117, no. 6: 4714–4758. 28272886 10.1021/acs.chemrev.6b00446

[mbo370116-bib-0034] Hoover, D. M. , K. R. Rajashankar , R. Blumenthal , et al. 2000. “The Structure of Human Beta‐Defensin‐2 Shows Evidence of Higher Order Oligomerization.” Journal of Biological Chemistry 275, no. 42: 32911–32918. 10906336 10.1074/jbc.M006098200

[mbo370116-bib-0035] Hosseinpour, S. , S. J. Roeters , M. Bonn , W. Peukert , S. Woutersen , and T. Weidner . 2020. “Structure and Dynamics of Interfacial Peptides and Proteins From Vibrational Sum‐Frequency Generation Spectroscopy.” Chemical Reviews 120, no. 7: 3420–3465. 31939659 10.1021/acs.chemrev.9b00410

[mbo370116-bib-0036] Hsu, S. T. , E. Breukink , E. Tischenko , et al. 2004. “The Nisin–Lipid II Complex Reveals a Pyrophosphate Cage That Provides a Blueprint for Novel Antibiotics.” Nature Structural & Molecular Biology 11, no. 10: 963–967. 10.1038/nsmb830 15361862

[mbo370116-bib-0037] Hu, J. , Y. Ye , X. Chen , L. Xiong , W. Xie , and P. Liu . 2022. “Insight Into the Pathogenic Mechanism of *Mycoplasma pneumoniae* .” Current Microbiology 80, no. 1: 14. 36459213 10.1007/s00284-022-03103-0 PMC9716528

[mbo370116-bib-0038] Hurd, A. J. 1999. “The Electrostatic Interaction Between Interfacial Colloidal Particles.” Journal of Physics A: Mathematical and General 18: L1055–L1060.

[mbo370116-bib-0039] Hussain, S. , and S. S. Maktedar . 2023. “Structural, Functional and Mechanical Performance of Advanced Graphene‐Based Composite Hydrogels.” Results in Chemistry 6: 101029.

[mbo370116-bib-0040] Hwang, P. M. , N. Zhou , X. Shan , C. H. Arrowsmith , and H. J. Vogel . 1998. “Three‐Dimensional Solution Structure of Lactoferricin B, an Antimicrobial Peptide Derived From Bovine Lactoferrin.” Biochemistry 37, no. 12: 4288–4298. 9521752 10.1021/bi972323m

[mbo370116-bib-0041] Jordan, J. B. , L. Poppe , M. Haniu , et al. 2009. “Hepcidin Revisited, Disulfide Connectivity, Dynamics, and Structure.” Journal of Biological Chemistry 284, no. 36: 24155–24167. 19553669 10.1074/jbc.M109.017764 PMC2782009

[mbo370116-bib-0042] Kaplan, C. W. , J. H. Sim , K. R. Shah , A. Kolesnikova‐Kaplan , W. Shi , and R. Eckert . 2011. “Selective Membrane Disruption: Mode of Action of C16G2, a Specifically Targeted Antimicrobial Peptide.” Antimicrobial Agents and Chemotherapy 55, no. 7: 3446–3452. 21518845 10.1128/AAC.00342-11 PMC3122425

[mbo370116-bib-0043] Kawulka, K. E. , T. Sprules , C. M. Diaper , et al. 2004. “Structure of Subtilosin A, a Cyclic Antimicrobial Peptide From *Bacillus subtilis* With Unusual Sulfur to α‐Carbon Cross‐Links: Formation and Reduction of α‐Thio‐α‐Amino Acid Derivatives.” Biochemistry 43, no. 12: 3385–3395. 15035610 10.1021/bi0359527

[mbo370116-bib-0044] Kufareva, I. , and R. Abagyan . 2012. “Methods of Protein Structure Comparison.” Methods in Molecular Biology (Clifton, NJ) 857: 231–257. 10.1007/978-1-61779-588-6_10 PMC4321859 22323224

[mbo370116-bib-0045] Kushibiki, T. , M. Kamiya , T. Aizawa , et al. 2014. “Interaction Between Tachyplesin I, an Antimicrobial Peptide Derived From Horseshoe Crab, and Lipopolysaccharide.” Biochimica et Biophysica Acta (BBA)—Proteins and Proteomics 1844, no. 3: 527–534. 24389234 10.1016/j.bbapap.2013.12.017

[mbo370116-bib-0046] Lassila, L. V. J. , S. Garoushi , J. Tanner , P. K. Vallittu , and E. Söderling . 2009. “Adherence of *Streptococcus mutans* to Fiber‐Reinforced Filling Composite and Conventional Restorative Materials.” Open Dentistry Journal 3: 227–232. 20148170 10.2174/1874210600903010227 PMC2817876

[mbo370116-bib-0047] Lemos, J. A. , S. R. Palmer , L. Zeng , et al. 2019. “The Biology of *Streptococcus mutans* .” Microbiology Spectrum 7, no. 1: 100171. 10.1016/j.bioflm.2023.100171. PMC6615571 30657107

[mbo370116-bib-0048] Loffredo, M. R. , A. Ghosh , N. Harmouche , et al. 2017. “Membrane Perturbing Activities and Structural Properties of the Frog‐Skin Derived Peptide Esculentin‐1a(1–21)NH2 and Its Diastereomer Esc(1–21)‐1c: Correlation With Their Antipseudomonal and Cytotoxic Activity.” Biochimica et Biophysica Acta (BBA)—Biomembranes 1859, no. 12: 2327–2339. 28912103 10.1016/j.bbamem.2017.09.009

[mbo370116-bib-0049] Makowski, M. , Í. C. Silva , C. Pais do Amaral , S. Gonçalves , and N. C. Santos . 2019. “Advances in Lipid and Metal Nanoparticles for Antimicrobial Peptide Delivery.” Pharmaceutics 11, no. 11: 588. 31717337 10.3390/pharmaceutics11110588 PMC6920925

[mbo370116-bib-0050] Mandard, N. , P. Sodano , H. Labbe , et al. 1998. “Solution Structure of Thanatin, a Potent Bactericidal and Fungicidal Insect Peptide, Determined From Proton Two‐Dimensional Nuclear Magnetic Resonance Data.” European Journal of Biochemistry 256, no. 2: 404–410. 9760181 10.1046/j.1432-1327.1998.2560404.x

[mbo370116-bib-0051] Manzer, H. S. , A. H. Nobbs , and K. S. Doran . 2020. “The Multifaceted Nature of Streptococcal Antigen I/II Proteins in Colonization and Disease Pathogenesis.” Frontiers in Microbiology 11: 602305. 33329493 10.3389/fmicb.2020.602305 PMC7732690

[mbo370116-bib-0052] Manzo, G. , P. M. Ferguson , C. K. Hind , et al. 2019. “Temporin L and Aurein 2.5 Have Identical Conformations but Subtly Distinct Membrane and Antibacterial Activities.” Scientific Reports 9, no. 1: 10934. 31358802 10.1038/s41598-019-47327-w PMC6662694

[mbo370116-bib-0053] Mariano, G. H. , L. G. Gomes de Sá , E. M. Carmo da Silva , et al. 2021. “Characterization of Novel Human Intragenic Antimicrobial Peptides, Incorporation and Release Studies From Ureasil‐Polyether Hybrid Matrix.” Materials Science & Engineering, C: Materials for Biological Applications 119: 111581. 33321627 10.1016/j.msec.2020.111581

[mbo370116-bib-0054] Mathavan, I. , S. Zirah , S. Mehmood , et al. 2014. “Structural Basis for Hijacking Siderophore Receptors by Antimicrobial Lasso Peptides.” Nature Chemical Biology 10, no. 5: 340–342. 24705590 10.1038/nchembio.1499 PMC3992131

[mbo370116-bib-0055] Matsumoto‐Nakano, M. 2018. “Role of *Streptococcus mutans* Surface Proteins for Biofilm Formation.” Japanese Dental Science Review 54, no. 1: 22–29. 29628998 10.1016/j.jdsr.2017.08.002 PMC5884221

[mbo370116-bib-0056] Meneses, I. H. C. , G. A. M. Sampaio , F. G. Carvalho , et al. 2020. “In Vivo Biocompatibility, Mechanical, and Antibacterial Properties of Cements Modified With Propolis in Different Concentrations.” European Journal of Dentistry 14, no. 1: 077–084. 10.1055/s-0040-1702255 PMC7079564 32168534

[mbo370116-bib-0057] Meng, X. Y. , H. X. Zhang , M. Mezei , and M. Cui . 2011. “Molecular Docking: A Powerful Approach for Structure‐Based Drug Discovery.” Current Computer Aided‐Drug Design 7, no. 2: 146–157. 21534921 10.2174/157340911795677602 PMC3151162

[mbo370116-bib-0058] Miller, 3rd, B. R. , T. D. McGee , J. M. Swails , N. Homeyer , H. Gohlke , and A. E. Roitberg . 2012. “MMPBSA.py: An Efficient Program for End‐State Free Energy Calculations.” Journal of Chemical Theory and Computation 8, no. 9: 3314–3321. 26605738 10.1021/ct300418h

[mbo370116-bib-0059] Montoya, C. , L. Roldan , M. Yu , et al. 2023. “Smart Dental Materials for Antimicrobial Applications.” Bioactive Materials 24: 1–19. 36582351 10.1016/j.bioactmat.2022.12.002 PMC9763696

[mbo370116-bib-0060] Moussa, D. G. , A. Fok , and C. Aparicio . 2019. “Hydrophobic and Antimicrobial Dentin: A Peptide‐Based 2‐tier Protective System for Dental Resin Composite Restorations.” Acta Biomaterialia 88: 251–265. 30753942 10.1016/j.actbio.2019.02.007 PMC6474255

[mbo370116-bib-0061] Namburu, J. R. , A. B. Rajendra Sanosh , C. S. Poosarla , S. Manthapuri , M. Pinnaka , and V. Baddam . 2022. “ *Streptococcus mutans*‐Specific Antimicrobial Peptide C16G2‐Mediated Caries Prevention: A Review.” Frontiers in Dentistry 19: 17. 36458273 10.18502/fid.v19i17.9963 PMC9675619

[mbo370116-bib-0062] Nguyen, V. S. , K. W. Tan , K. Ramesh , F. T. Chew , and Y. K. Mok . 2017. “Structural Basis for the Bacterial Membrane Insertion of Dermcidin Peptide, DCD‐1L.” Scientific Reports 7, no. 1: 13923. 29066724 10.1038/s41598-017-13600-z PMC5654962

[mbo370116-bib-0063] Oh, D. , Y. Kim , S. Y. Shin , J. H. Kang , K. S. Hahm , and K. L. Kim . 1999. “NMR Structural Characterization of Cecropin A(1–8)–Magainin 2(1–12) and Cecropin A (1–8)–Melittin (1–12) Hybrid Peptides.” Journal of Peptide Research 53, no. 5: 578–589. 10.1034/j.1399-3011.1999.00067.x 10424354

[mbo370116-bib-0064] Panday, S. K. , and E. Alexov . 2022. “Protein–Protein Binding Free Energy Predictions With the MM/PBSA Approach Complemented With the Gaussian‐Based Method for Entropy Estimation.” ACS Omega 7, no. 13: 11057–11067. 35415339 10.1021/acsomega.1c07037 PMC8991903

[mbo370116-bib-0065] Pantsar, T. , and A. Poso . 2018. “Binding Affinity via Docking: Fact and Fiction.” Molecules 23, no. 8: 1899. 30061498 10.3390/molecules23081899 PMC6222344

[mbo370116-bib-0066] Petit, V. W. , J. L. Rolland , A. Blond , et al. 2016. “A Hemocyanin‐Derived Antimicrobial Peptide From the Penaeid Shrimp Adopts an Alpha‐Helical Structure That Specifically Permeabilizes Fungal Membranes.” Biochimica et Biophysica Acta (BBA)—General Subjects 1860, no. 3: 557–568. 26708991 10.1016/j.bbagen.2015.12.010

[mbo370116-bib-0067] Pettersen, E. F. , T. D. Goddard , C. C. Huang , et al. 2004. “UCSF Chimera—A Visualization System for Exploratory Research and Analysis.” Journal of Computational Chemistry 25, no. 13: 1605–1612. 15264254 10.1002/jcc.20084

[mbo370116-bib-0068] Pillong, M. , J. A. Hiss , P. Schneider , et al. 2017. “Rational Design of Membrane‐Pore‐Forming Peptides.” Small 13, no. 40: 1701316. 10.1002/smll.201701316 28799716

[mbo370116-bib-0069] Powers, J.‐P. S. , A. Rozek , and R. E. W. Hancock . 2004. “Structure–Activity Relationships for the β‐Hairpin Cationic Antimicrobial Peptide Polyphemusin I.” Biochimica et Biophysica Acta (BBA)—Proteins and Proteomics 1698, no. 2: 239–250. 15134657 10.1016/j.bbapap.2003.12.009

[mbo370116-bib-0070] Pratap, B. , R. K. Gupta , B. Bhardwaj , and M. Nag . 2019. “Resin Based Restorative Dental Materials: Characteristics and Future Perspectives.” Japanese Dental Science Review 55, no. 1: 126–138. 31687052 10.1016/j.jdsr.2019.09.004 PMC6819877

[mbo370116-bib-0071] Pronk, S. , S. Páll , R. Schulz , et al. 2013. “GROMACS 4.5: A High‐Throughput and Highly Parallel Open Source Molecular Simulation Toolkit.” Bioinformatics 29, no. 7: 845–854. 23407358 10.1093/bioinformatics/btt055 PMC3605599

[mbo370116-bib-0072] Rifai, E. A. , V. Ferrario , J. Pleiss , and D. P. Geerke . 2020. “Combined Linear Interaction Energy and Alchemical Solvation Free‐Energy Approach for Protein‐Binding Affinity Computation.” Journal of Chemical Theory and Computation 16, no. 2: 1300–1310. 31894691 10.1021/acs.jctc.9b00890 PMC7017367

[mbo370116-bib-0073] Runge, S. , H. Thøgersen , K. Madsen , J. Lau , and R. Rudolph . 2008. “Crystal Structure of the Ligand‐Bound Glucagon‐Like Peptide‐1 Receptor Extracellular Domain.” Journal of Biological Chemistry 283, no. 17: 11340–11347. 18287102 10.1074/jbc.M708740200

[mbo370116-bib-0074] Saini, R. S. , R. I. H. Binduhayyim , V. Gurumurthy , et al. 2024a. “Dental Biomaterials Redefined: Molecular Docking and Dynamics‐Driven Dental Resin Composite Optimization.” BMC Oral Health 24, no. 1: 557. 38735940 10.1186/s12903-024-04343-1 PMC11089745

[mbo370116-bib-0075] Saini, R. S. , R. I. H. Binduhayyim , V. Gurumurthy , et al. 2024b. “In Silico Assessment of Biocompatibility and Toxicity: Molecular Docking and Dynamics Simulation of PMMA‐Based Dental Materials for Interim Prosthetic Restorations.” Journal of Materials Science: Materials in Medicine 35, no. 1: 28. 38833196 10.1007/s10856-024-06799-7 PMC11150300

[mbo370116-bib-0076] Sanusi, Z. K. , and K. A. Lobb . 2022. “Insights into the Dynamics and Binding of Two Polyprotein Substrate Cleavage Points in the Context of the SARS‐CoV‐2 Main and Papain‐Like Proteases.” Molecules 27, no. 23: 8251. 36500348 10.3390/molecules27238251 PMC9740519

[mbo370116-bib-0077] Schrödinger, L. 2020. The PyMOL Molecular Graphics System. www.pymol.org.

[mbo370116-bib-0078] Skrtic, D. , J. M. Antonucci , and D. W. Liu . 2006. “Ethoxylated Bisphenol Dimethacrylate‐Based Amorphous Calcium Phosphate Composites.” Acta Biomaterialia 2, no. 1: 85–94. 16701862 10.1016/j.actbio.2005.10.004 PMC1839056

[mbo370116-bib-0079] Sparks, R. P. , J. L. Jenkins , and R. Fratti . 2019. “Use of Surface Plasmon Resonance (SPR) to Determine Binding Affinities and Kinetic Parameters Between Components Important in Fusion Machinery.” Methods in Molecular Biology 1860: 199–210. 30317506 10.1007/978-1-4939-8760-3_12 PMC8489108

[mbo370116-bib-0080] Terwilliger, T. C. , L. Weissman , and D. Eisenberg . 1982. “The Structure of Melittin in the Form I Crystals and Its Implication for Melittin's Lytic and Surface Activities.” Biophysical Journal 37, no. 1: 353–361. 7055627 10.1016/S0006-3495(82)84683-3 PMC1329151

[mbo370116-bib-0081] Tian, S. , H. Sun , P. Pan , et al. 2014. “Assessing an Ensemble Docking‐Based Virtual Screening Strategy for Kinase Targets by Considering Protein Flexibility.” Journal of Chemical Information and Modeling 54, no. 10: 2664–2679. 25233367 10.1021/ci500414b

[mbo370116-bib-0082] Tian, W. , C. Chen , X. Lei , J. Zhao , and J. Liang . 2018. “CASTp 3.0: Computed Atlas of Surface Topography of Proteins.” Nucleic Acids Research 46, no. 1: W363–W367. 29860391 10.1093/nar/gky473 PMC6031066

[mbo370116-bib-0083] Valdés‐Tresanco, M. S. , M. E. Valdés‐Tresanco , P. A. Valiente , and E. Moreno . 2021. “gmx_MMPBSA: A New Tool to Perform End‐State Free Energy Calculations With GROMACS.” Journal of Chemical Theory and Computation 17, no. 10: 6281–6291. 34586825 10.1021/acs.jctc.1c00645

[mbo370116-bib-0084] Van Landuyt, K. L. , J. Snauwaert , M. Peumans , J. De Munck , P. Lambrechts , and B. Van Meerbeek . 2008. “The Role of HEMA in One‐Step Self‐Etch Adhesives.” Dental Materials 24: 1412–1419. 18433860 10.1016/j.dental.2008.02.018

[mbo370116-bib-0085] Viana de Freitas, T. , U. Karmakar , A. G. Vasconcelos , et al. 2023. “Release of Immunomodulatory Peptides at Bacterial Membrane Interfaces as a Novel Strategy to Fight Microorganisms.” Journal of Biological Chemistry 299, no. 4: 103056. 36822328 10.1016/j.jbc.2023.103056 PMC10074799

[mbo370116-bib-0086] Wang, G. 2008. “Structures of Human Host Defense Cathelicidin LL‐37 and Its Smallest Antimicrobial Peptide KR‐12 in Lipid Micelles.” Journal of Biological Chemistry 283, no. 47: 32637–32643. 18818205 10.1074/jbc.M805533200

[mbo370116-bib-0087] Wang, G. , Y. Li , and X. Li . 2005. “Correlation of Three‐Dimensional Structures With the Antibacterial Activity of a Group of Peptides Designed Based on a Nontoxic Bacterial Membrane Anchor.” Journal of Biological Chemistry 280, no. 7: 5803–5811. 15572363 10.1074/jbc.M410116200

[mbo370116-bib-0088] Xie, S. X. , L. Song , E. Yuca , et al. 2020. “Antimicrobial Peptide–Polymer Conjugates for Dentistry.” ACS Applied Polymer Materials 2, no. 3: 1134–1144. 33834166 10.1021/acsapm.9b00921 PMC8026165

[mbo370116-bib-0089] Yang, J. , X. Liang , F. Liu , Y. Biao , and J. He . 2024. “Comparing Properties of Urethane Dimethacrylate (UDMA) Based 3D Printing Resin Using N‐Acryloylmorpholine (ACMO) and Triethylene Glycol Dimethacrylate (TEGDMA) Separately as Diluents.” Journal of Macromolecular Science, Part B 64, no. 9: 1005–1021.

[mbo370116-bib-0090] Yuan, C. , R. Xing , J. Cui , W. Fan , J. Li , and X. Yan . 2023. “Multistep Desolvation as a Fundamental Principle Governing Peptide Self‐Assembly Through Liquid–Liquid Phase Separation.” CCS Chemistry 6, no. 1: 255–265.

[mbo370116-bib-0091] Yuan, Z. , X. Chen , S. Fan , et al. 2024. “Binding Free Energy Calculation Based on the Fragment Molecular Orbital Method and Its Application in Designing Novel SHP‐2 Allosteric Inhibitors.” International Journal of Molecular Sciences 25: 671. 10.3390/ijms25010671. 38203841 PMC10779950

[mbo370116-bib-0092] Yuet, P. K. , and D. Blankschtein . 2010. “Molecular Dynamics Simulation Study of Water Surfaces: Comparison of Flexible Water Models.” Journal of Physical Chemistry B 114: 13786–13795. 20939552 10.1021/jp1067022

[mbo370116-bib-0093] Zangger, K. , R. Gößler , L. Khatai , K. Lohner , and A. Jilek . 2008. “Structures of the Glycine‐Rich Diastereomeric Peptides Bombinin H2 and H4.” Toxicon 52, no. 2: 246–254. 18586045 10.1016/j.toxicon.2008.05.011

[mbo370116-bib-0094] Zhang, J. , Y. Yang , Y. Chen , et al. 2024. “A Review of New Generation of Dental Restorative Resin Composites With Antibacterial, Remineralizing and Self‐Healing Capabilities.” Discover Nano 19, no. 1: 189. 39570468 10.1186/s11671-024-04151-0 PMC11582236

[mbo370116-bib-0095] Zhang, Q. Y. , Z. B. Yan , Y. M. Meng , et al. 2021. “Antimicrobial Peptides: Mechanism of Action, Activity and Clinical Potential.” Military Medical Research 8, no. 1: 48. 34496967 10.1186/s40779-021-00343-2 PMC8425997

[mbo370116-bib-0096] Zhou, J.‐S. , H.‐L. Wen , and M.‐J. Yu . 2024. “Mechanism Analysis of Antimicrobial Peptide NoPv1 Related to Potato Late Blight Through a Computer‐Aided Study.” International Journal of Molecular Sciences 25, no. 10: 5312. 38791351 10.3390/ijms25105312 PMC11121460

[mbo370116-bib-0097] Zhu, J. , N. Avakyan , A. Kakkis , et al. 2021. “Protein Assembly by Design.” Chemical Reviews 121, no. 22: 13701–13796. 34405992 10.1021/acs.chemrev.1c00308 PMC9148388

